# The translation initiation factor eIF2α regulates lipid homeostasis and metabolic aging

**DOI:** 10.1111/acel.14348

**Published:** 2024-10-15

**Authors:** Haipeng Huang, Yilie Liao, Ning Li, Xingfan Qu, Chaocan Li, Jiaqi Hou

**Affiliations:** ^1^ School of Life Sciences Tsinghua University Beijing China; ^2^ Institute of Molecular Medicine, College of Future Technology Peking University Beijing China; ^3^ Tianjin Key Laboratory of Aquatic Science and Technology, School of Environmental and Municipal Engineering Tianjin Chengjian University Tianjin China; ^4^ State Key Laboratory of Environmental Criteria and Risk Assessment Chinese Research Academy of Environmental Sciences Beijing China

**Keywords:** aging, eIF2α, ISR, lipid metabolism, mitochondria, translation

## Abstract

Aging is usually accompanied by excessive body fat gain, leading to increased susceptibility to comorbidities. This study aimed to explore an unexpected function for the eukaryotic initiation factor‐2α (eIF2α) during aging. Reducing the eIF2α dose led to a reconfiguration of the metabolic equilibrium, promoting catabolism, facilitating lipolysis, and decreasing body fat accumulation while maintaining healthy glucose and lipid metabolism during aging. Specifically, eIF2α enhanced the expression of distinct messenger RNAs encoding mitochondrial electron transport chain proteins at the translation level. The mitochondrial respiration increased in eIF2α heterozygotes, even during aging. Deceleration of translation was demonstrated as a conserved mechanism for promoting longevity across various species. Our findings demonstrated that the restriction of translation by reducing eIF2α expression could fend off multiple tissue damage and improve metabolic homeostasis during aging. Hence, eIF2α was a crucial target for benefiting mammalian aging achieving delayed mammalian aging.

## Graphical abstract

We established a mouse model with suppressed translation. The eIF2α heterozygotes alleviated the integrated stress response in multi‐organs. Reducing eIF2α resulted in a translational regulation that increased multi‐ETC proteins, concomitantly decreased Raptor, and suppressed mTORC1. Lowering eIF2α led a reconfiguration of the metabolic equilibrium, promoting catabolism and facilitating lipolysis, preventing excessive accumulation of body fat while maintaining healthy glucose and lipid metabolism in aging.

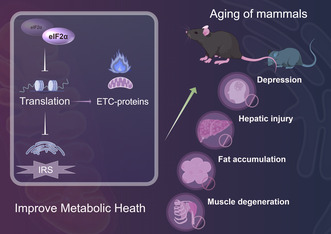

**Abbreviations**
AAs: amino acids
ALT: alanine transaminase
AST: aspartate aminotransferase
CHO: cholesterol
DNL: de novo lipogenesis
ECM: elevated cross maze
eEFs: elongation factors
eIF2α: eukaryotic initiation factor 2α
eIFs: eukaryotic initiation factors
ERs: endoplasmic reticulum stress
ETC: electron transport chain
FAO: fatty acid oxidation
GTT: glucose tolerance test
IL6: interleukin 6
ISR: integrated stress response
ITT: insulin tolerance test
mTOR: mechanistic target of rapamycin
mTORC1/2: mechanistic target of rapamycin complex 1/2
OFT: open field test
Ps: polysomes
TEM: transmission electron microscope.
TGs: triglycerides
TNFα: tumor necrosis factor α
WB: western blot
WT: wild‐type

## 1 INTRODUCTION

The translation is accomplished through the collaboration of small and large ribosomal subunits with mRNA, assisted by eukaryotic initiation factors (eIFs) (Jackson et al., 2010) and elongation factors (eEFs), ensuring both swiftness and precision. Appropriate translation flux coupled folding processes are required for the maintenance of proteostasis (Anisimova et al., 2018; Derisbourg, Hartman, & Denzel, 2021; Kennedy et al., 2014; Klaips et al., 2018; Steffen & Dillin, 2016). The eukaryotic translation initiation factor 2α (eIF2α) is involved in the forming a ternary complex with GTP and initiator tRNA (Kimball, 1999). During aging, integrated stress response (ISR) is triggered in response to various cellular stresses, such as endoplasmic reticulum stress (ERs). Consequently, unfolded proteins accumulate in endoplasmic reticulum, disrupting the protein homeostasis network (Klaips et al., 2018; Kourtis & Tavernarakis, 2011; Kyriakakis et al., 2015; Steffen & Dillin, 2016). The phosphorylation of the eIF2α (p‐eIF2α) responded to the ISR signal significantly reduces ternary complex formation and attenuates global protein synthesis (Adomavicius et al., 2019; Jiang et al., 2003; Kimball et al., 1998). The ISR plays a pivotal role in maintaining organismal resilience, and the key node of the ISR is the eIF2α that controls global protein synthesis as the central regulator of mRNA translation initiation (Merrick & Pavitt, 2018).

The mechanistic target of rapamycin (mTOR) orchestrates cellular growth and metabolism in eukaryotes, governing essential cellular processes such as protein synthesis (Saxton & Sabatini, 2017). The deficiency of TOR in the nematode *Caenorhabditis elegans* more than doubles its natural lifespan (Vellai et al., 2003). Inhibition of mTOR with the approved therapeutic rapamycin was recognized to promote health and longevity in diverse model organisms (Mannick & Lamming, 2023). However, directly regulating translation by reducing the levels of ribosomal proteins, ribosomal‐protein S6 kinase or translation initiation factors also increased the lifespan of *C. elegans* (Hansen et al., 2007). Specifically, loss of eIF4E reduces global protein synthesis, protects from oxidative stress, and extends lifespan in *C. elegans*; knockdown of TOR further increases the longevity of the mutants (Syntichaki et al., 2007). Thus, translation inhibition might serve as one of the downstream mechanisms by which mTOR affects lifespan.

During aging, mice exhibit elevated levels of eIF2α phosphorylation in liver, muscle, and kidney (Chalil et al., 2015; Ladiges et al., 2000), a phenomenon that has also been observed in various of other lower organisms (Baker et al., 2012; Brown et al., 2014; Derisbourg, Wester, et al., 2021; Kulalert et al., 2017; Kulalert & Kim, 2013). This increase aims to inhibit over‐translation during aging by reducing the working eIF2α levels. The modulation of eIF2α activity can influence lifespan. In *C. elegans*, increased lifespan is mediated by mitochondrial dysfunction depending on eIF2α phosphorylation (Baker et al., 2012). The phosphorylation of eIF2α in neurons is a key mediator of the state transition to promote survival (Kulalert et al., 2017; Kulalert & Kim, 2013). However, the use of *C. elegans* in investigating the role of eIF2α during aging is relatively limited. A mutated mouse, with a mutation at the eIF2α phosphorylation site (Ser51Ala) rendering it unresponsive to ISR signaling and translation regulation, succumbed to hypoglycemia associated with defective gluconeogenesis within 18 h after birth (Scheuner et al., 2001). However, continuous activation of ISR was also lethal (Harding et al., 2009). Overall, numerous mouse models have been used to manipulate ISR and translation flux. However, whether genetic manipulation of translation affects mammalian aging remains unknown. Furthermore, distinct from the consistently observed advantageous effect on the longevity of translation suppression in lower animal models, whether this is beneficial in mammals remains unclear. A development of a corresponding mouse model that can restrict excessive translation and mitigate endoplasmic integrated stress has yet to be developed.

Aging is associated with an increase in abdominal obesity, which is a significant contributor to insulin resistance and the metabolic syndrome. Preclinical studies have indicated that complex lipids may play a role in regulating longevity, as most tissues exhibit pronounced lipid accumulation with aging (Janssens et al., 2024; Tsugawa et al., 2024). In human subjects, each 0.27 kg increment in visceral fat has been linked to a cognitive aging of approximately 0.7 years (Mina et al., 2023). Overweight is a risk factor for accelerated aging and reduced life expectancy. Therefore, weight management and treatment of obesity play a crucial role in combating the process of accelerated aging. Additionally, addressing insulin resistance is essential for mitigating age‐related effects (Chen et al., 2023). Ribosomal damage induced by ROS could lead obesity and aging‐related metabolic diseases (Snieckute et al., 2023). Our laboratory generated an eIF2α+/− mouse model which demonstrating half the ribosome eIF2α protein levels of wild‐type (WT) mice. IRS was effectively alleviated in eIF2α+/− mice coupled with downregulated protein synthesis. Furthermore, eIF2α+/− mice showed lower body weight from adulthood through old age. We comprehensively analyzed various physiological indicators in mice regarding the aging process. Our objective was to employ this innovative mouse model to investigate the impact of eIF2α heterozygosity on mammalian health throughout the aging trajectory.

## 2 RESULTS

### 2.1 eIF2α heterozygous diminished weight gain by inhibiting fat accumulation

**FIGURE 1:**
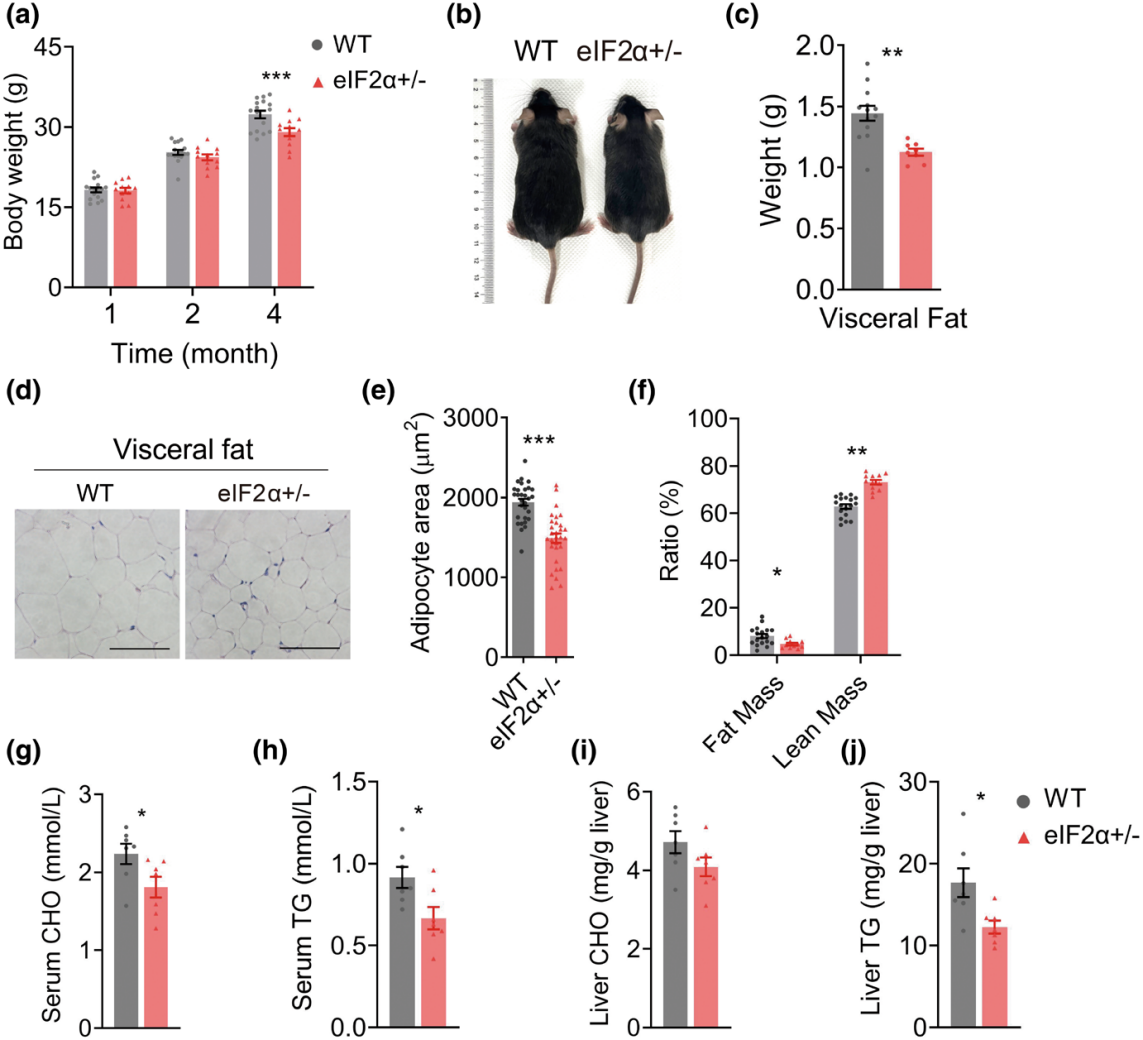
eIF2α heterozygous diminished weight gain by inhibiting fat accumulation. (a) Body weight of WT (*n* = 13 ~ 16) and eIF2α+/− (*n* = 12 ~ 13) mice at indicated age. (b) Representative images of WT and eIF2α+/− mice (male) at the age of 5 months old. (c) Weight of visceral fat. (d, e) Representative images of H&E staining visceral fat sections (Scale bar, 200 μm) (d), measurement of adipocyte area (*n* = 27 ~ 30, from four images of two mice for each group, 4 months old) (e). (f) Body composition examination at the age of 4 months of WT (*n* = 18) and eIF2α+/− (*n* = 12) mice. (g–j) Measurement of serum cholesterol (CHO, *n* = 7 vs. 7) (g) and triglyceride (TG, *n* = 7 vs. 7) (h), liver cholesterol (CHO, *n* = 7 vs. 7) (i), and triglyceride (TG, *n* = 7 vs. 7) (j) in WT and eIF2α+/− mice at age of 4 months old. All mice used in the above experiments were male. Data are represented as mean ± SEM. *p < 0.05, **p < 0.01, and ***p < 0.001. See also Figure S1.

Sequence insertion suppressed the expression of the targeted allele (Figure S1a). We found that eIF2α+/− mice at the age of 4 months weighed significantly less than WT mice from the same litter (Figure 1a), while presenting smaller body sizes (Figure 1b). The absence of variation in tibia length suggested no alterations in developmental progression (Figure S1d,e). Consistent with these findings, eIF2α+/− mice showed a lower visceral fat weight than WT mice (Figure 1c), and the adipose tissue exhibited a smaller size of adipocytes (Figure 1d,e). The body composition analysis revealed that eIF2α+/− mice exhibited a lower fat composition than WT mice (Figure 1f). Correspondingly, a higher percentage of lean mass composition was observed. However, we found no differences in body weight or size of female eIF2α+/− and WT mice of the same age. The eukaryotic initiation factors are involved in lipid metabolism regulation (Birkenfeld et al., 2011; Conn et al., 2021). So, we measured the levels of triglycerides (TGs) and cholesterol (CHO) in the serum and liver. The serum TG and CHO, and liver TG levels significantly decreased in eIF2α+/− mice (Figure 1g–j). Our data collectively indicated that a distinct underlying mechanism played a crucial role in age‐related fat accumulation via eIF2α, concurrently reducing both serum and liver lipid levels. However, the lower body weight induced by eIF2α+/− was not observed in female mice at 4 months of age (Figure S1f). This may be attributed to the fact that females generally have lower body weight compared to males, and as a result, the regulatory effect of eIF2α+/− has not yet been significant in females (Figure S1g).

### 2.2 Reducing eIF2α level decreased translation and promoted catabolism

**FIGURE 2:**
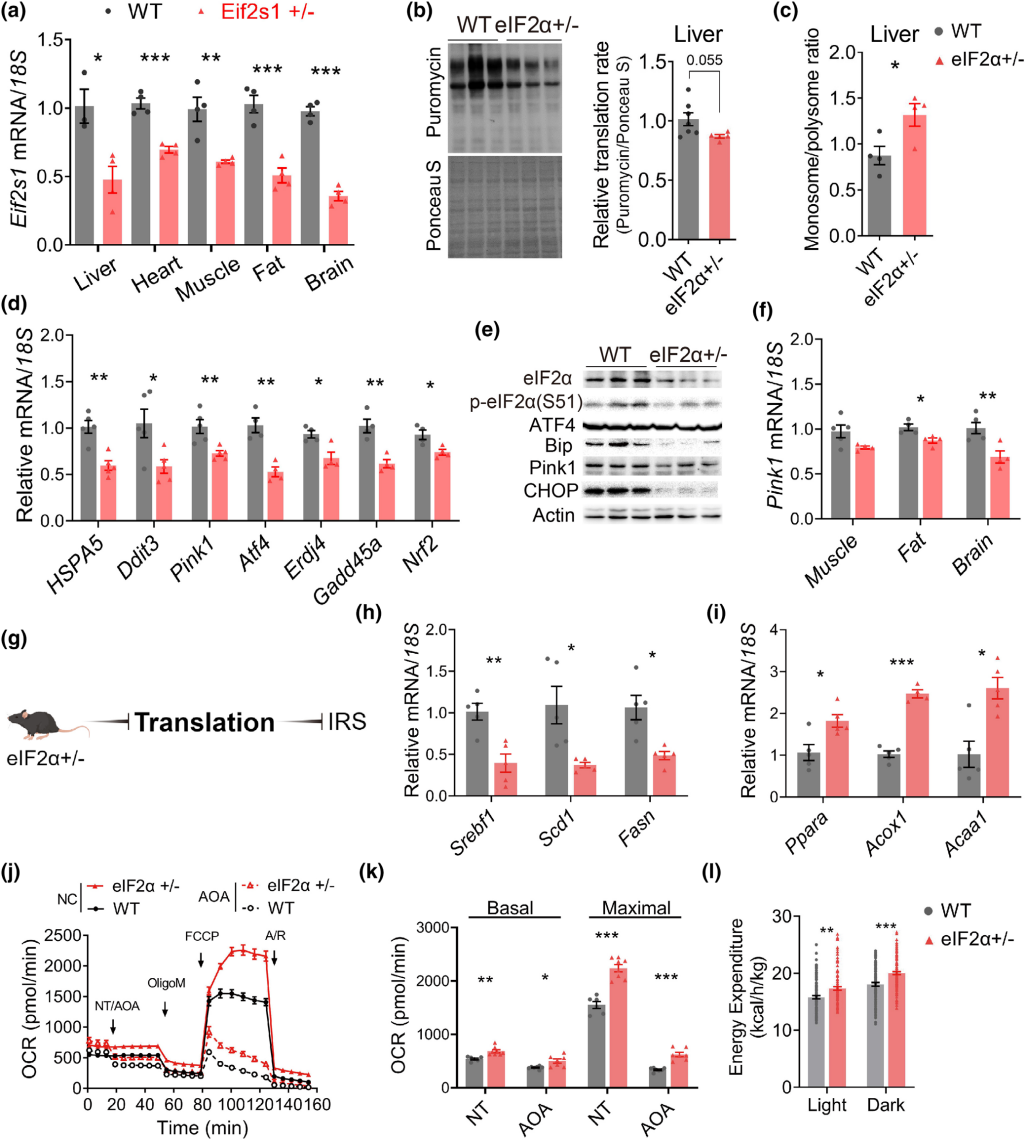
Lower eIF2α reduced translation and promoted catabolism. (a) Measurement of the transcript levels of *Eif2s1* in multi‐tissues. Normalized to *18S* ribosomal RNA (*n* = 3–5). Mice at the age of 4 months old. (b) Western blot of translating peptides in the liver (left); quantify of translational flux, the level of puromycin labeling was normalized to total protein, and a representative experiment with *n* = 5 per condition was normalized to the mean value of WT mice (right). (c) Polysome isolation and quantification of the ratio of monosomes (80S) to polysomes (*n* = 4 mice for each group). (d) Measurement of the transcript levels of genes in ISR‐related pathways in mouse liver samples. Normalized to 18S ribosomal RNA (*n* = 5). (e) Western blot of ISR‐related proteins in the liver. (f) Measurement of the transcript levels of *Pink1* in multi‐tissues. Normalized to *18S* ribosomal RNA (*n* = 4–5). (g) Schematic representation illustrating the attenuation of translation and alleviation of ISR through the reduction in eIF2α levels. (h, i) RT‐qPCR examinations of gene expression, de novo lipogenesis (DNL) related genes (h), fatty acid oxidation (FAO) related genes (i) of WT (*n* = 5) and eIF2α+/− (*n* = 5) mice (4 months old). (j, k) Mitochondrial respiration measurement of hepatocyte (4‐month‐old mice), oxygen consumption rate (OCR) was calculated to represent respiration level (j), OCR inhibited by AOA (inhibitor of the transaminase process) indicates amino acid (AA)‐contributed respiration (k). (l) Metabolic cage experiment, measurement of mice energy expenditure of WT and eIF2α+/− mice (4 months old) (*n* = 8 vs. 8). The mice utilized in the aforementioned experiments were 4–5‐month‐old male mice. Data are represented as mean ± SEM. *p < 0.05, **p < 0.01, and ***p < 0.001. See also Figures S2 and S3.

Next, we delved into how eIF2α+/− reduced body fat composition. The model was applied as a 50% *Eif2s1* expression eIF2α+/− model (Figure 2a and Figure S2a). As a pivotal regulator of metabolic homeostasis, the liver coordinates lipoprotein synthesis and transportation, as well as facilitates lipid oxidation to generate energetic metabolites for other tissues (Kulalert & Kim, 2013). We employed an in vivo translation labeling method to annotate the translational levels in the liver (Figure S2b). The mice were fed with a controlled diet to minimize the effects of energy levels on translation (Figure S2b). The nascent peptide chains during translation were labeled with puromycin, and the puromycin signal on the label was detected using western blot (WB) (Figure 2b). The WB analysis indicated a lower translation flux of about 15% in eIF2α+/− mice (Figure 2b). We also applied a polysome profiling technique that separated and quantified free ribosomal subunits, monosomes (mRNA with one associated ribosome), and polysomes (mRNA with two or more associated ribosomes) using optical density measurements over a sucrose density gradient. Remarkably, we observed a shift from polysomes to monosomes in eIF2α+/− mice, suggesting that more cytosolic mRNAs were translated by a single ribosome instead of multiple ribosomes following the reduction in the eIF2α levels (Figure S2c). WT mice displayed an increased polysome proportion. The calculation of the ratio of monomers versus polysomes showed a higher ratio of eIF2α+/− mice (Figure 2c), which further supported the in vivo labeling results. Correspondingly, multiple ISR‐related signals in the liver were significantly reduced in eIF2α+/− mice (Figure 2d,e), suggesting that the restrictions on translation relieved ERs and ISR. PTEN‐induced kinase 1 (Pink1) was expression decreased in liver and many other tissues of eIF2α+/− mice (Figure 2d–f). The levels of ISR markers, *Atf4* and *Erdj4*, were also measured in the muscle, fat, and brain (Figure S2d,e). Consistent with liver, ISR was suppressed in these tissues, particularly the brain (Figure 2f and Figure S2d,e). Overall, the reduction in eIF2α level significantly decreased the translation, and ISR was alleviated in various organs of mice (Figure 2g). ERs plays a crucial role in the coordination of lipid metabolism and metabolic reprogramming (Moncan et al., 2021). Hence, we detected the lipid metabolism pathways in the liver. The expression levels of de novo lipogenesis (DNL) genes, *Srebf1*, *Scd1*, and *Fasn* significantly decrease (Figure 2h). In contrast, the expression levels of fatty acid oxidation (FAO)‐related genes, specifically *Ppara*, *Acox1* and *Acaa1*, were markedly upregulated (Figure 2i). These results suggested inhibition of DNL and promotion of FAO in the liver, consistent with a lower liver weight (Figure S2f,g). However, no significant difference was observed in the expression of glycolysis‐related genes (Figure S3a), indicating the specificity of eIF2α+/− in regulating lipid metabolic pathways.

Mitochondrial FAO is the major pathway for the degradation of fatty acids and is essential for maintaining energy homeostasis. Subsequently, we employed isolated primary hepatocytes to assess energy production. The phosphorylation of AMPK was inhibited in eIF2α+/− hepatocytes, indicating a corresponding increased ATP level (Figure S3b,c). The mitochondrial respiratory capacity was then examined, revealing a significant augmentation in eIF2α+/− hepatocytes (Figure 2j,k). Moreover, no significant difference in the number of mitochondria and mitochondrial ROS level were observed (Figure S3d,e). Amino acids (AAs) serve as crucial substrates for mitochondrial respiration (Li & Hoppe, 2023), and inhibiting translation would result in substantial AAs available for energy production in mitochondria. Aminooxy acetate (AOA) was used to inhibit transaminase so as to investigate whether the enhanced mitochondrial respiration in eIF2α+/− hepatocytes was attributed to the increased supply of AAs. We found that AA‐fueled respiration accounted for approximately 50% of the increase in eIF2α+/− hepatocytes (Figure S3f). This implies that, besides modulating AAs flux through translational inhibition, eIF2α+/− enhanced mitochondrial respiration through other potential mechanisms.

Reduced ISR was detected in multiple tissues. Hence, we hypothesized that translation and catabolism regulated by eIF2α+/− were not limited to the liver and might be systemic. We next investigated the metabolic rate in mice. eIF2α+/− mice exhibited elevated energy expenditure and metabolic rate, which were observed throughout the light and dark cycles (Figure 2l and Figure S3g). Also, higher activity levels were noted in eIF2α+/− mice (Figure S3h). These results suggested that reduced eIF2α dose led to an increase in body catabolism in mice. Pink1 level was conservatively reduced in multiple tissues in eIF2α+/− mice (Figure 2d–f), suggesting high quality and robust mitochondria in eIF2α+/− mice.

### 2.3 eIF2α+/− reprogramed the translational regulation of electron transport chain proteins

**FIGURE 3:**
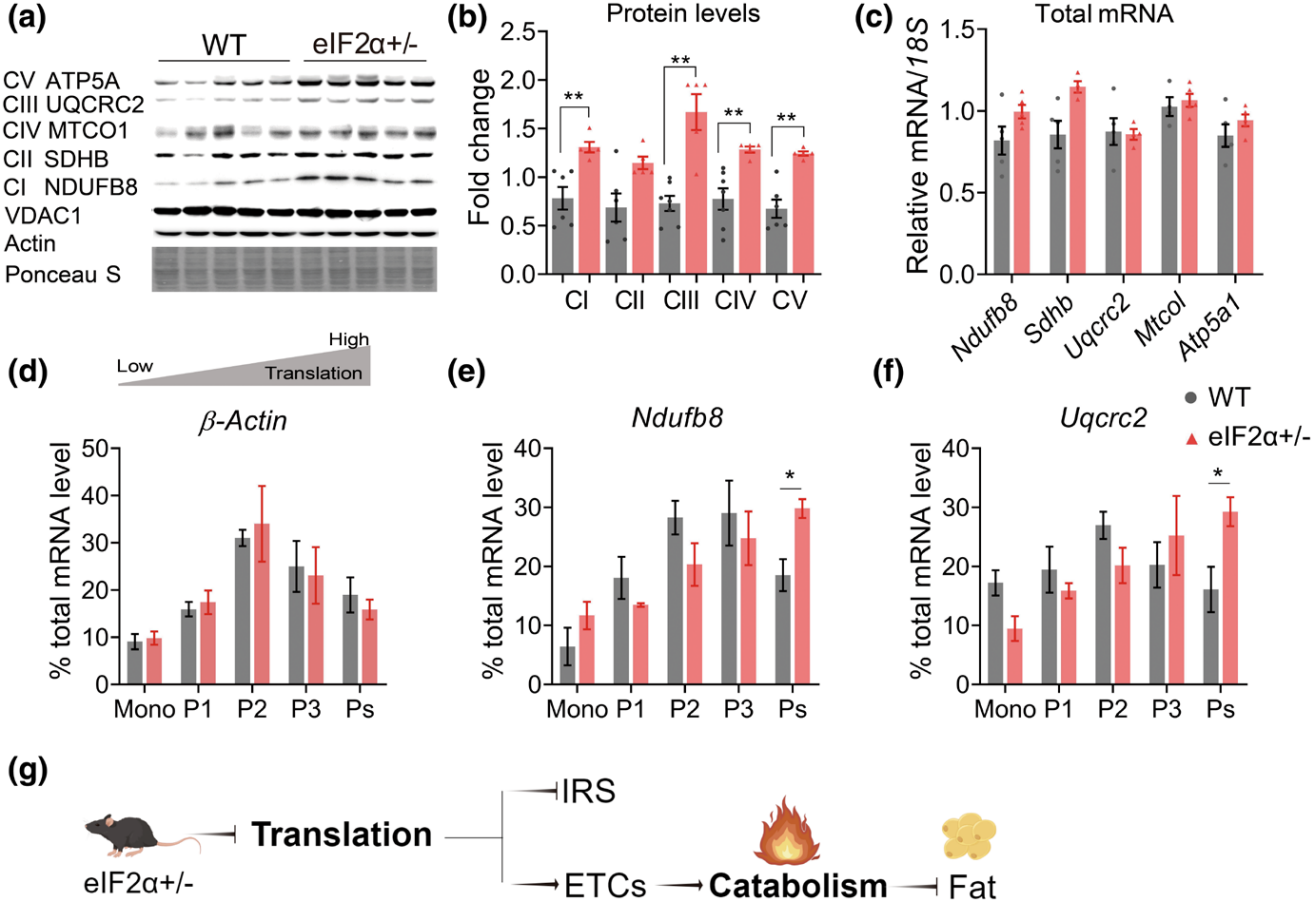
eIF2α+/− reprogramed translational regulation of ETC proteins. (a, b) Representative western blot (a) and densitometry results (b) for ETC proteins in the liver of WT and eIF2α+/− mice. (c) RT‐qPCR examinations of electron transport chain (ETC) genes expression of WT (*n* = 5) and eIF2α+/− (*n* = 5) mice (4 months old). (d–f) Representative qPCR analysis of genes isolated from sucrose gradient fractions of WT or eIF2α+/− livers. The 40s, 60s, and 80s fractions were combined as a “mono” group. Values correspond to the percentage of total mRNA across fractions indicated in Figure S2c, fractions qPCR analysis of *Actin* (d), ETC genes (e, f) (*n* = 3 for each group). (g) Schematic representation illustrating the promotion of mitochondrial vitality to increase catabolism, which then consume more fat in eIF2α+/− mice. All mice used in the above experiments were male. Data are represented as mean ± SEM. *p < 0.05, **p < 0.01. See also Figure S4.

We had found that about half of the enhanced mitochondrial respiration was not attributed to AAs contributions (Figure S3f). Then, we postulated that eIF2α might potentially enhance mitochondrial performance through alternative mechanisms. We directly evaluated the levels of representative proteins involved in the mitochondrial electron transport chain (ETC), revealing a significant elevation in the levels of the 5 ETC proteins in eIF2α+/− mice (Figure 3a,b). The elevations were subsequently demonstrated in other tissues of muscle and heart, as well as in primary hepatocytes (Figure S4a,b), but not observed in the brain. Furthermore, mRNA levels of these ETC proteins were measured no significant change (Figure 3c). We hypothesized that eIF2α might increase the level of ETC proteins through translational regulation. Using the ribosomal sucrose fractions isolated in previous experiments (Figure S2c), we extracted relevant mRNA and assessed the gene transcript levels. Using *Actin* as a control, we observed no significant disparity in the distribution of ribosomes between the two groups (Figure 3d). Four of the ETC genes showed changes in the ribosomal distribution. More transcripts were enriched in the Polysomes (Ps) group of the eIF2α+/− mice (Figure 3e,f and Figure S4c–e). These results indicate that while the downregulation of eIF2α inhibits overall translation, variations in the levels of eIF2α, a critical factor in translational regulation, result in translational adjustments. The upsurge in mitochondrial ETC protein levels is attributed to eIF2α‐mediated translational regulation.

As the mammalian/mechanistic target of rapamycin complex 1 (mTORC1) is a master regulator of numerous cellular processes implicated in mitochondrial metabolism. We then detected the mTORC1 signal, which decreased in the livers of eIF2α+/− mice (Figure S4f). Meanwhile, a difference in the mTORC2 signal was not observed (Figure S4f). Thus, enhanced mitochondrial respiration in the eIF2α+/− mouse model was not caused by mTORC2 signaling. We detected a decrease in the level of Raptor protein in eIF2α+/−, which was consistent with the fact that Raptor was detected with fewer transcripts enriched in the Ps group of the eIF2α+/− mice (Figure S4g). However, the total mRNA level did not change (Figure S4h). These results suggested a translational regulation of *Raptor* through change in eIF2α dose. Collectively, these data demonstrated a translational upregulation of mitochondrial ETC proteins in eIF2α+/− mice, thereby increasing ETC protein levels, and improving mitochondria respiration (Figure 3g). The inhibition of protein degradation by MG132 had no effects on the elevated ETC levels in eIF2α+/− mice (Figure S3b), suggesting that is was not related to the protein degradation pathway. Furthermore, RNA interference to silence eIF2α expression in C2C12 and HeLa cell lines resulted in successful upregulation of ETC proteins, leading to an increase in mitochondrial respiration (data not shown).

### 2.4 eIF2α+/− mitigated metabolic disorders and hepatic injury associated with aging

**FIGURE 4:**
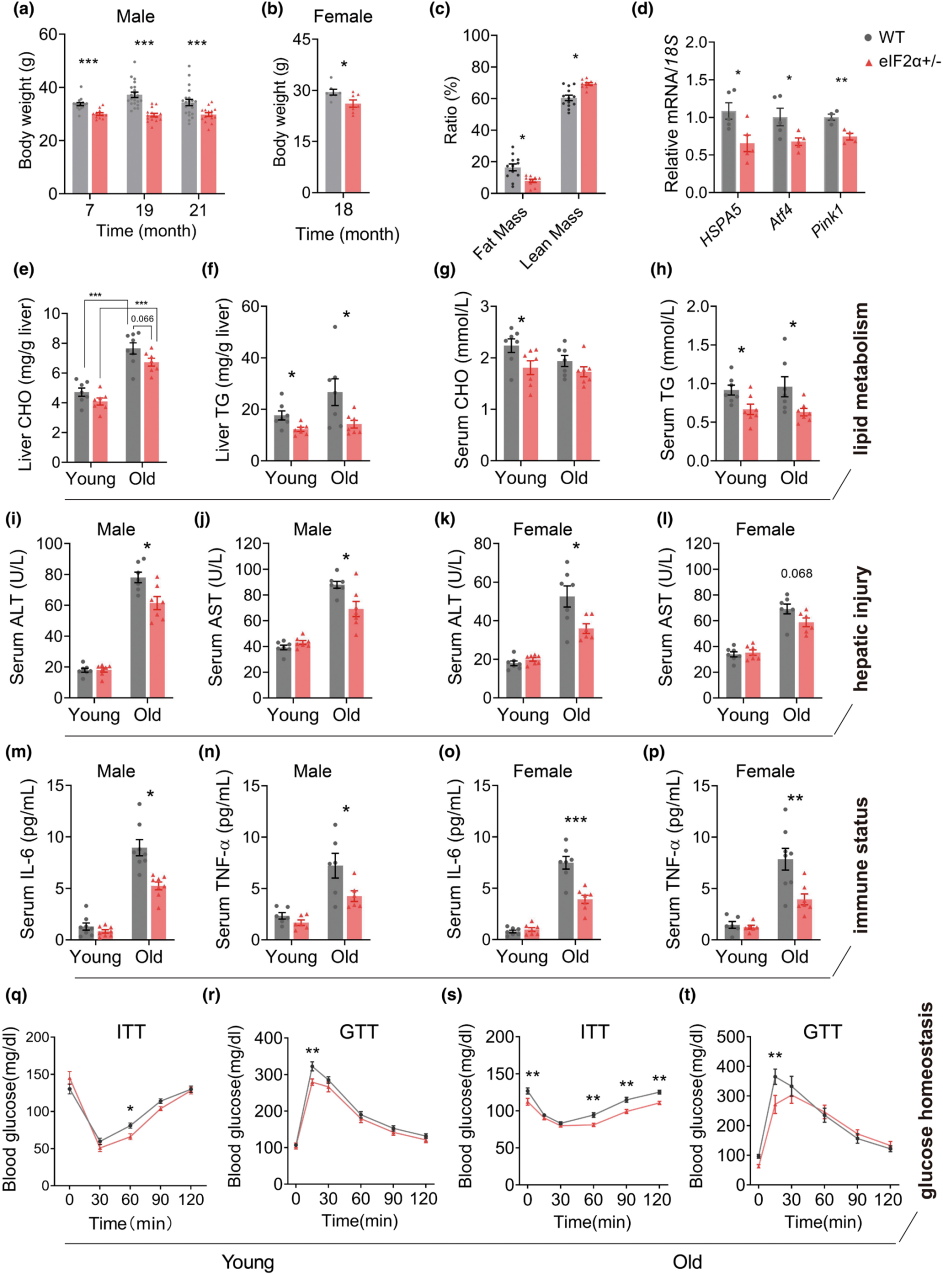
Mitigated metabolic disorders associated with aging in eIF2α+/− mice. (a) Body weight of male WT (*n* = 15–20) and eIF2α+/− (*n* = 12–16) mice at indicated age. (b) Body weight of female WT and eIF2α+/− mice (*n* = 7 for each group) at 18 months old. (c) Body composition examination at the age of 19 months of male WT (*n* = 13) and eIF2α+/− (*n* = 10) mice. (d) Measurement of the transcript levels of genes in UPR‐related pathways in the liver of 24‐month‐old male mice. Normalized to *18S* ribosomal RNA (*n* = 4–5). (e–h) Measurement of liver CHO (e) and TG (f), serum CHO (g) and TG (h) in young and aged male mice, WT and eIF2α+/− (*n* = 7–8, for 4‐ and 24‐month‐old mice). (i–l) Serum ALT and AST levels of male (i, j) and female (k, l) mice, WT (*n* = 6–8) and eIF2α+/− (*n* = 6–8) mice at the age of 4 and 24 months old. (m–p) Serum IL‐6 and TNF‐α levels of male (m, n) and female (o, p) mice, WT (*n* = 6–8) and eIF2α+/− (*n* = 6–8) mice at the age of 4 and 24 months old. (q–t) Insulin tolerance test (ITT) (q) and glucose tolerance test (GTT) (r) of WT (*n* = 11) and eIF2α+/− (*n* = 8) mice at the age of 4 months old; ITT (s) and GTT (t) of WT (*n* = 9) and eIF2α+/− (*n* = 11) mice at the age of 21 months old. Data are represented as mean ± SEM. *p < 0.05, **p < 0.01, and ***p < 0.001. See also Figure S5.

Subsequently, we examined the significance of the eIF2α+/− in the process of biological aging. eIF2α+/− mice maintained a consistently lower body weight from middle to old age and exhibited resistance to age‐related body weight gain (Figure 4a). Moreover, a relatively lower body weight was found during aging eIF2α+/− female mice (Figure 4b), suggesting a consistent regulatory mechanism of decreased eIF2α expression. The lower body weight of eIF2α+/− mice was resulted from a lower fat mass percentage compared with WT mice (Figure 4c). In these aging mice, ISR was found consistently suppressed of eIF2α+/− (Figure 4d).

We then examined multiple metabolic markers in these aging mice. The TG was found significantly lower in the liver and serum of eIF2α+/− mice; however, the changes in CHO were not significant (Figure 4e–h). Then, age‐related serum risk factors were assessed in male and female mice. The serum levels of alanine transaminase (ALT) and aspartate aminotransferase (AST) were all remarkably elevated during aging (Figure 4i–l). eIF2α+/− mice showed decrease in serum ALT and AST levels compared with WT mice, indicating amelioration of liver injury. Aging is always accompanied by immune disorders (Ellulu et al., 2017; Hotamisligil, 2006). We found a decrease in the levels of interleukin 6 (IL6) and the tumor necrosis factor‐α (TNFα) associated with aging in both male and female eIF2α+/− mice (Figure 4m–p). These findings proved that eIF2α+/− mice exhibited remission to both hyperactivation of immunity and liver damage during aging.

Moreover, the age‐related elevation of serum insulin levels was mitigated in eIF2α+/− mice (Figure S5a). eIF2α+/− improved insulin sensitivity in the insulin tolerance test (ITT) and glucose tolerance in the glucose tolerance test (GTT) both in young and aged mice (Figure 4q–t). These results indicate the beneficial effects of altered metabolic homeostasis in eIF2α+/− mice, which effectively improve glucose metabolism in aging mice. Overall, our data suggest that reducing eIF2α expression enhances mitochondrial respiration, regulates metabolic homeostasis, and reduces body fat accumulation during aging.

### 2.5 Effects of reducing eIF2α expression on multiple age‐associated morbidities

**FIGURE 5:**
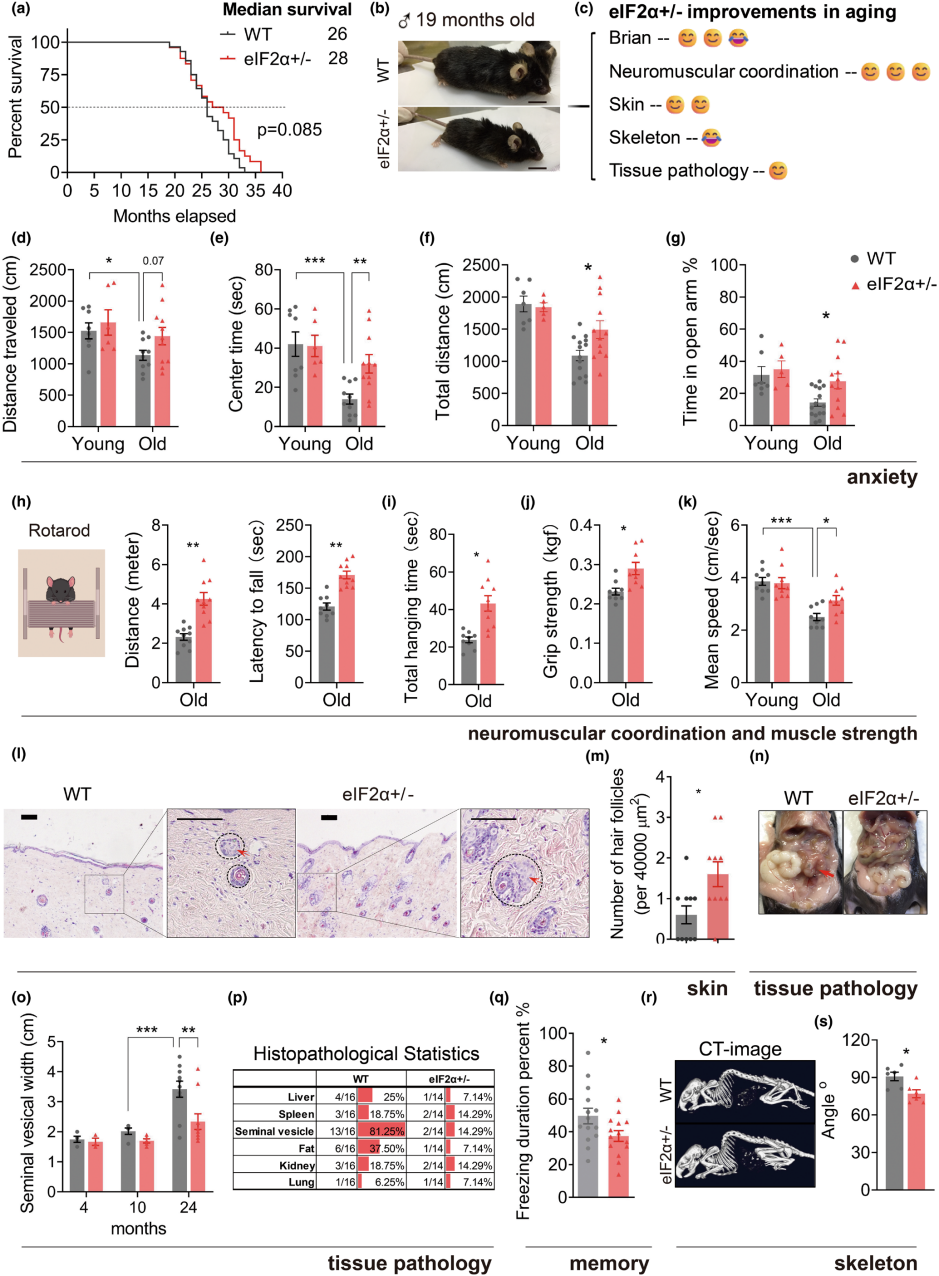
Effects of lowering eIF2α to multiple age‐associated morbidities. (a) Lifespan curves of male WT (*n* = 28) and eIF2α+/− (*n* = 24) mice, data were collected up to the age of 24 months. Log‐rank (Mantel‐Cox) test was used to compare the survival curves. (b) Representative images of WT and eIF2α+/− mice at age of 19 months old. (c) Schematic representation illustrating the effect of eIF2α+/− on the functional decline of tissues associated with aging. (d, e) Open field experiments were conducted on 4 months old (*n* = 8 vs. 6) and 22 months old (*n* = 10 vs. 11) WT and eIF2α+/− mice, total traveled distance (d), and time spent in the center (e). (f, g) Elevated cross mazes were conducted on 4 months old (*n* = 8 vs. 5) and 22 months old (*n* = 14 vs. 11) WT and eIF2α+/− mice, total traveled distance (f), and time spent in the open arm (g). (h–j) Neuromuscular and muscle strength: Rotarod test and the measured running distance and total running time on the rotating rod (h); wire hang time (i); grip‐strength tests (j). (k) The speed of locomotor activity of mice in the open field was quantified. (l) Skin and hair follicles of 24‐month‐old aging mice, micrograph of a section through the skin, showing hair follicles (dark purple circular structures). Representative images of H&E staining (Scale bars, 100 μm). (m) Quantitative analysis of the number of hair follicles per unit area in skin sections. (n, o) Representative images of seminal vesicle (n) and measurements of vesicle width at the age of 4, 10, and 24 months old of WT (*n* = 4–10) and eIF2α+/− (*n* = 4–9) mice (o). (p) Histopathological statistics of 24‐month‐old aging mice, WT (*n* = 15–20) and eIF2α+/− (*n* = 13–20). (q) Fear‐conditioned memory test of the WT (*n* = 14) and eIF2α+/− (*n* = 14) mice at the 22 months old, fear memory remind test. (r, s) CT imaging was performed to visualize the mouse skeleton of WT and eIF2α+/− mice on 4 months old (*n* = 5 for each group) and 23 months old (*n* = 6 for each group), representative images of mice skeleton (r), spinal curvature was quantified respectively (s). Data are represented as mean ± SEM. *p < 0.05, **p < 0.01, and ***p < 0.001. See also Figure S5.

We then assessed multiple senescence‐associated phenotypes. Applying the translation labeling approach, we found that eIF2α+/− maintained a lower translation level than WT mice during aging (Figure S5b). eIF2α+/− male mice did not survive significant longer compared with WT mice (Figure 5a). Of these old mice, eIF2α+/− mice exhibited a leaner build and a shinier coat (Figure 5b and Figure S5c). Anxiety‐related exploratory behaviors were significantly reduced in aging. To test the impact of lower eIF2α on post aging exploration ability, we utilized the open field test (OFT) and elevated cross maze (ECM) test to measure the exploratory behaviors of mice. Compared with the younger group, the aged mice showed a remarkable decrease in exploration activity within an unfamiliar environment; this decline was mitigated in eIF2α+/− mice compared to WT mice (Figure 5d,e and Figure S5d). Similarly, the residence time of the open arm (ECM) was significantly higher in aged eIF2α+/− mice compared with WT mice (Figure 5f,g). These results indicated that aged eIF2α+/− mice had better exploratory motor abilities and lower anxiety levels. An analysis of the neuromuscular functions showed that the distance run in the rotarod test and the latency to fall increased in eIF2α+/− mice (Figure 5h), whereas the total hanging time in the wire hang test increased (Figure 5i). The grip‐strength tests revealed that eIF2α+/− mice had higher muscle strength compared with WT mice (Figure 5j). We also calculated the movement speed of mice in the open field experiment; eIF2α+/− mice showed significant higher mean speed (Figure 5k). Consistent with the decreased immune factor levels in the serum in eIF2α+/− mice (Figure 4m–p), the infiltration of immune cells in the liver reduced in eIF2α+/− mice (Figure S5e). Additionally, the hair were darker and more densely packed in eIF2α+/− mice (Figure S5c). The slices of the skin revealed that eIF2α+/− mice had a higher number of hair follicles, with a clearer and better‐organized arrangement of cells within the hair follicle structure (Figure 5l,m and Figure S5f). Aging is characterized by the concurrent degeneration and hyperplasia of multiple organs, and the proliferation of tumors (Schumacher et al., 2021). Remarkably, seminal vesicle hyperplasia occurs with high frequency in mice from middle to old age (Figure 5n and Figure S5g), which was significantly inhibited in eIF2α+/− mice (Figure 5o). Moreover, we observed and quantified the incidence of various tissue lesions and tumors (Figure S5h,i). eIF2α+/− mice exhibited a reduction in the occurrence of diverse tissue tumors and hyperplasia, particularly seminal vesicle hyperplasia (from 81.25% to 14.29%) and lipoma (from 37.5% to 7.14%) (Figure 5p).

However, negative effects of reducing eIF2α were also shown during aging. A fear‐conditioned memory test was employed to assess the memory of mice (Figure S5j). No difference in freezing time was detected in the training test (Figure S5j). The fear conditioning test revealed a decreased fear memory in eIF2α+/− mice (Figure 5q). The angle of spinal curvature was determined as shown in Figure S5k; the more curved the spine, the smaller this angle became. Computed tomography scans revealed a higher degree of curvature in eIF2α+/− mice than in WT mice (Figure 5r,s).

### 2.6 Relieved recession and injury of mitochondria of eIF2α+/− mice during aging

**FIGURE 6:**
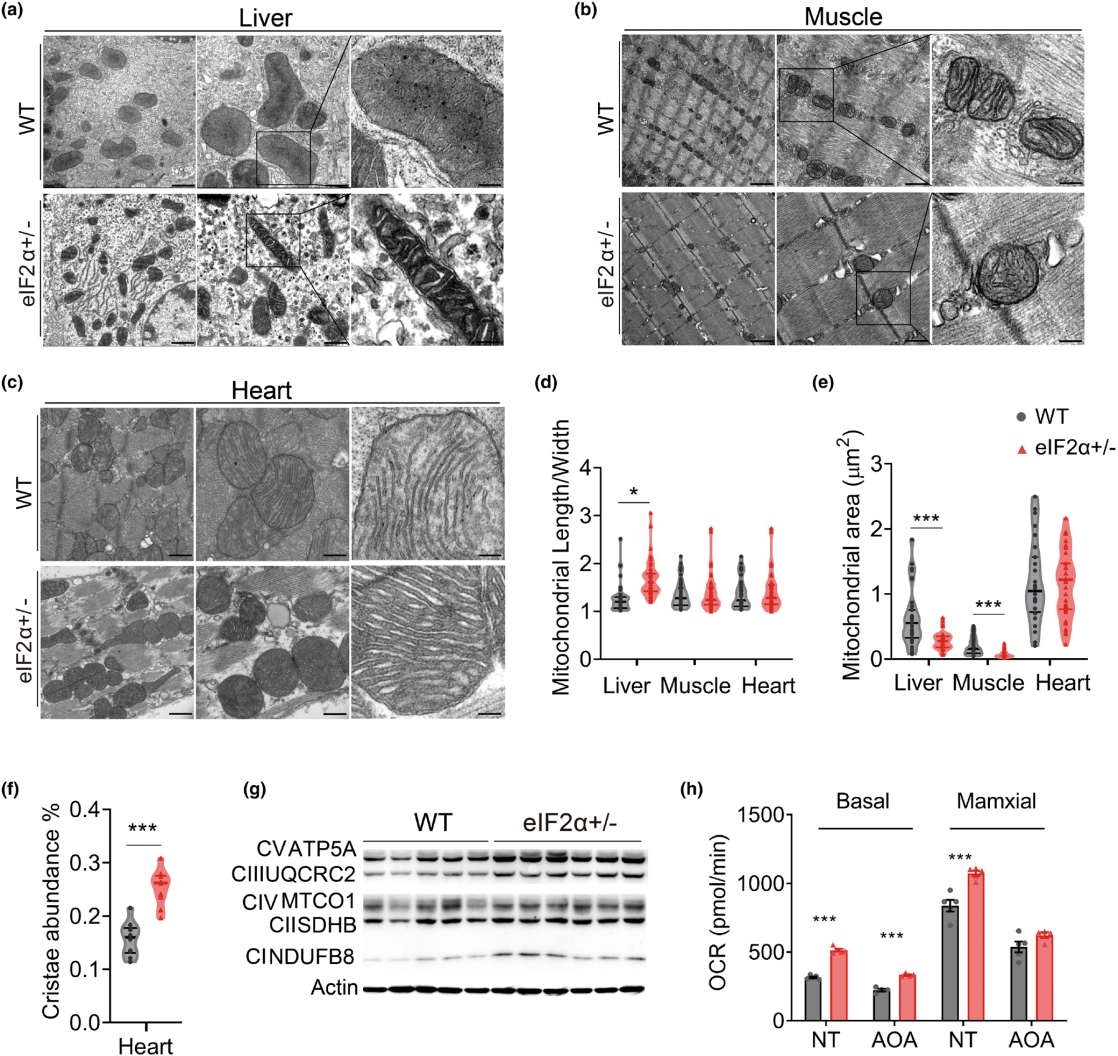
Relieved recession and injury of mitochondria of eIF2α+/− mice in aging. (a–c) Ultrastructure of mitochondria in the liver (a), muscle (b), and heart (c) of 24‐month‐old mice (Scale bars, left to right, 1 μm, 500 nm, 150 nm). (d–f) Quantification of aspect ratio to represent mitochondrial morphology (d), mitochondrial area (*n* > 50 for liver and muscle, *n* > 30 for heart) (for each tissue, mitochondrial quantified from 6 ultrastructure images of two mice) (e), quantification of mitochondrial cristae abundance for heart (f). (g) Western blot of ETC proteins in the liver of 24‐month‐old mice. (h) Measurement of mitochondrial respiration in hepatocytes of mice at 22 months old. Data are represented as mean ± SEM. *p < 0.05, ***p < 0.001. See also Figure S6.

We found that reducing eIF2α level promoted mitochondrial respiration, reshaped metabolic homeostasis, and enhanced lipolysis. Consequently, the mice resisted the accumulation of body fat during aging, benefiting metabolic health. However, whether the eIF2α‐regulated mitochondrial respiration was maintained until after aging and whether it caused more severe damage to the mitochondria in eIF2α+/− mice remained unclear. Using a transmission electron microscope (TEM), we directly detected the ultramicrostructure of mitochondria in the liver, muscle, and heart (Figure 6a–c). Accumulating evidence suggested an age‐related increase in mitochondrial swelling. Also, the inner ridge architecture of mitochondria underwent degeneration, resulting in a tendency for mitochondria to assume a spherical shape (Amorim et al., 2022). We observed that the mitochondria in the livers of WT mice exhibited a morphology characterized by short, plump columns and spheres. The statistical analysis showed a significantly higher length‐to‐width ratio in eIF2α+/− mice (Figure 6d and Figure S6a–c). Moreover, the mitochondrial area was significantly lower in eIF2α+/− mice, in both liver and muscle tissues (Figure 6e). These findings suggested that decreased eIF2α expression protected mitochondria, inhibiting age‐related mitochondrial damage and swelling. The linear ridge serves as the primary site of respiration of mitochondria. We endeavored to quantify the area occupied by the cristae to assess whether eIF2α conferred a protective effect on mitochondrial ridge structure (Figure S6d). We found a dramatic elevation (~30%) of cristae content in the hearts of eIF2α+/− mice (Figure 6f). Collectively, these data showed that eIF2α regulated a mitochondrial protection mechanism, preserved the integrity of the mitochondrial cristae structure during aging, and inhibited mitochondrial swelling.

Consistent with the TEM results, the levels of several representative mitochondrial ETC proteins increased in eIF2α+/− mice, consistent with the results obtained in young mice (Figure 6g). Next, we examined the respiratory capacity of mitochondria in aging mice. The mitochondria in the hepatocytes of eIF2α+/− mice maintained a higher respiratory capacity compared with that of WT mice, even after blocking of AAs under basal conditions (Figure 6h). qPCR analysis indicated higher expression levels of FAO genes in the liver of eIF2α+/− mice (Figure S6e), while PINK1 pathway was still inhibited in eIF2α+/− mice (Figure S6f), suggesting a protective effect on mitochondria in the context of aging. The data collectively demonstrated that the preservation of complete structure and better respiratory activity in mitochondria was safeguarded by eIF2α and sustained throughout the aging process.

## 3 DISCUSSION

In this study, we employed a novel animal model to detect various physiological effects of the diminished eIF2α expression in mammalian senescence. Our study encompassed establish a mouse model with suppressed translation, eIF2α heterozygote. We conducting the polysome isolation and the translation flux labeling experiments, monitored various physiological markers, examined mitochondrial activity at the cellular level through primary cell systems, and tracked alterations in these parameters throughout the natural aging process in mice. Through these approach, we identified an eIF2α‐meditated mechanism whereby the translation suppression and enhanced mitochondrial respiratory capacity were communicated to increase catabolism, thereby suppressing excessive fat accumulation and benefiting metabolic health during aging.

We unexpectedly discovered that diminished eIF2α levels enhanced metabolic fitness and, in turn, prevented fat gain while aging. We revealed a unique mechanism for eIF2α‐dependent translation in the controlling mitochondrial respiratory capacity and quality control. By inhibiting ribosomal proteins, eIFs/eEFs or ribosomal RNA expression was used to investigate the impact of protein synthesis on multiple biological processes (Brown et al., 2014; Chalil et al., 2015; Chiocchetti et al., 2007; Derisbourg, Wester, et al., 2021; Hansen et al., 2007; Hetz & Saxena, 2017; Kourtis & Tavernarakis, 2011; Kyriakakis et al., 2015). However, studies investigating mammalian aging using the aforementioned methodologies were scarce. The heterozygous Eif2s1 ± mouse model generated by the International Mouse Phenotyping Consortium also displayed reduced body weight, which is in line with our results. The experimental duration was limited to the period from birth until 5 months for mice, leading to an inability to detect aging‐related phenotypes. We performed multi‐level physiological monitoring using eIF2α+/− mice and compared age‐related phenotypes. Comprehensive experiment results were obtained across different age groups of mice, indicating the persistent efficacy of the intervention in mitigating the aging process (Figure 4e–t). This study proved the benefits of endogenous translation limitation by directly regulating translation elements to mammalian aging, which are now can be considered as effects conserved across species in different genetic backgrounds. Our data supported that the modulation by eIF2α played a role in establishing a link between protein synthesis (Figure 2b,c) and catabolism (Figure 2k,l). On the one hand, the repression of translation allowed more AAs to be retained and made available to the mitochondria; on the other hand, ETC protein levels were elevated through the translational regulation by eIF2α (Figure S7). We have also employed RNA interference to silence eIF2α expression in C2C12 and HeLa cell lines, resulting in successful upregulation of ETC proteins, and leading increase in mitochondrial respiration (data not shown). Thus, the robust mitochondria in eIF2α+/− elevated fatty acid consumption. Collectively, these results supported the beneficial impact of translational restriction on both aging and metabolic processes.

The faithful translation of mRNA requires precise coordination, despite the high precision mechanism of the translation engine, translation errors are pervasive (Skariah & Todd, 2021; Song et al., 2021). Cells need active regulatory mechanisms to deal with these errors. Conceptually, slowing protein translation might allow mRNA to be translated into protein with higher fidelity or to fold more accurately (Drummond & Wilke, 2009). Our results showed that decreased eIF2α dose was advantageous for mitigating the ISR pathway, resulting in increased overall fitness of protein homeostasis during aging (Figure 2d–f and Figure S7). The eIF2α played a central role in the ISR pathways. The intracellular state of reducing the protein level of eIF2α (eIF2α ±) was likely to mimic the activated state of the ISR downstream branching pathway regulated by eIF2α. Thus, cellular damage signals such as ERs decreased, favoring intracellular protein homeostasis. ISR inhibition ameliorated pathology in mouse models of the progeroid Down syndrome (Zhu et al., 2019) and Alzheimer's disease (Oliveira et al., 2021). Moreover, the beneficial effects of ISR inhibition were reported in Huntington's disease (Krzyzosiak et al., 2018), ALS (Das et al., 2015), and multiple sclerosis (Way et al., 2015). We found better exploratory motor abilities and lower anxiety levels in the aged eIF2α+/− mice. This provided new evidence for mitigating the ISR on neurological function in the context of aging. The haem‐regulated inhibitor activating compounds 1‐((1,4‐trans)‐4‐aryloxycyclohexyl)‐3‐arylureas increased p‐eIF2α levels, downstream of the ISR and slowed cancer cell proliferation (Chen et al., 2013). Our statistical results suggested that reducing eIF2α expression reduced the rate of organ lesions in multiple tissues, including the development of hepatic tumors.

The eIF2α+/− mice exhibited varying effects on organ functions. Specifically, the downregulation of eIF2α led to no impact on the heart and muscle weight (Figure S2g). Similarly, the protective impact of lower eIF2α in different organs varies during aging. We began to detect aging‐related phenotypes in mice from 19 months of age. The aging phenotypes were collected over a total of 5 months. The metabolically beneficial results by eIF2α+/− were detected at 19, 21, and 24 months of age (Figure 4c–t). Reduction in eIF2α significantly enhanced exploratory behavior in 22‐month‐old mice and significantly improved locomotor abilities associated with neuromuscular regulation in 23‐month‐old mice (Figure 5d–k). As neuronal memory consolidation relied heavily on rapid, high‐throughput protein synthesis (Gold, 2008; Mac Callum et al., 2014), thus which was inhibited in eIF2α+/− mice of 22 months old (Figure 5q). These findings (Figure 5c) highlighted the pronounced organ‐specific effects of eIF2α+/− regulation, which is also determined by the eIF2α distinctive function in the translation and ISR pathway. On the contrary, the results of the lower eIF2α effects on aging are better supported by the fact that aging phenotype detection experiments have been carried out across almost the entire aging age range, rather than at a single time point.

There is strong preclinical evidence in mice that rapamycin can extend lifespan. There is rapidly growing interest in using mTOR inhibitors to promote healthy aging and to treat, delay, or reverse numerous age‐related diseases (Mannick & Lamming, 2023). However, hyperlipidemia and hyperglycemia have been observed in rodents treated with rapamycin, although mice are less susceptible to developing hyperlipidemia‐induced cardiovascular disease than humans (Firpi et al., 2004; Johnston et al., 2008; Krueger et al., 2016; Trelinska et al., 2015). An 8‐week long randomized clinical trial of rapamycin at 1 mg per day on 25 older adults (aged between 70 and 95) showed an increase in glycated hemoglobin levels and a 40% rise in triglyceride levels compared to the placebo group (Kraig et al., 2018). In contrast to the effects on metabolism produced by inhibition of mTOR, direct reduction in eIF2α to inhibit translation produces favorable effects on glucose metabolism and lipid metabolism homeostasis during aging (Figure 4q–t and Figure S5a). This might have resulted from the fact that mTORC2 was not significantly regulated in our model. The survival curve did not show a significant lifespan extension through the reduction in eIF2α in our experiments. However, the improvement of glucose/lipid metabolism, the benefits of metabolism, inhibition of proliferative lesions, and oncogenesis in multiple tissues highlighted the possibility of achieving healthy aging by inhibiting eIF2α expression. The targeting of eIF2α should be prioritized as a crucial objective in the pursuit of delaying mammalian aging.

## 4 MATERIALS AND METHODS

### 4.1 Mouse husbandry and experimentation

All the animal husbandry and experimental procedures were in strict accordance with the guidelines approved by the Institutional Animal Care and Use Committee (IACUC) of Tsinghua University.

The eIF2α+/− mice model was generated by BIOCYTOGEN Bioscience (Beijing, China). Loxp and other sequences were inserted to block the expression of the mutant chain (Figure S1a). Genotyping primers were designed to amplify the inserted Loxp sequence. eIF2α+/− mice were backcrossed for 10 generations to C57Bl/6J mice (Figure S1b,c). Genotyping primers: *eIF2α*‐A1‐LoxpF: F: 5′‐CATGATAGTAAAATGTTGGGTGCTGG‐3′, *eIF2α*‐A2‐LoxpR: R: 5′‐ACTATCTATAAAGTGACGTGAGGCCTAAG‐3′. WT: 251 bp, Mut: 341 bp.

All the mice were housed in a specific pathogen‐free (SPF) facility at Tsinghua Laboratory Animal Research Center under a 12/12‐h light–dark cycle and controlled temperature (25 ± 1°C) with free access to water and food. Nutrient composition of animal feed used in mouse feeding was listed (Figure S1d), protein accounts for 19.3% of the total nutrients, and fat accounts for 13.4% of the total nutrients.

### 4.2 Chemicals and antibodies

The following chemicals were used in this study: Protease and phosphatase inhibitor cocktails (Pierce, Cat.no. 78441); TRIzol reagent (Life Technologies, Cat.no. 15596018); FastKing RT Kit (KR116‐02, Tiangen); qPCR SYBR green mix (Abm, Cat.no. MasterMix‐LR); Prestained protein marker (Thermo Scientific, Cat.no. 26616); ECL (Merck Milipore, Cat.no. WBKL50500); PCR primers were synthesized in Sangon Biotech; BCA (Solarbio, Cat.no. PC0021); RIPA (Radio‐Immunoprecipitation Assay) Lysis Buffer (Thermo Scientific, Cat.no. 89900); Puromycin (Solarbio, Cat.no. P8230‐25); Ponceaus S (Solarbio, Cat.no. P8330); Glucose (Amresco, Cat.no. 0188‐500G). Antibodies used are listed as follows: Mouse anti‐ eIF2α (Cell Signaling Technology, Cat.no. 2103S); Rabbit anti‐HistonH3 (Proteintech, Cat.no. 17168‐1‐AP); Mouse anti‐Puromycin (Merck Millipore, Cat.no. MABE343); Rabbit anti‐BiP (Cell Signaling Technology, Cat.no. 3177S); Rabbit anti‐Pink1 (Proteintech, Cat.no. 23274‐1‐AP); Mouse anti‐β‐Actin (Abgent, Cat.no. AM1021B). Rabbit anti‐phospho‐eIF2α (Ser51) (Cell Signaling Technology, Cat.no. 3597S); OXPHOS Rodent WB antibody cocktail (including ATP5A, UQCRC2, SDHB, mtCO1) (Abcam, Cat.no. ab110413).

### 4.3 Reverse transcribed quantitative polymerase chain reaction (RT‐qPCR)

Total RNA was extracted from liver tissues with TRIzol reagent (Life Technologies, Cat.no. 15596018) according to the manufacturer's recommendations. An aliquot of 0.5 μg RNA was reverse transcribed to cDNA by FastKing RT Kit (KR116‐02, Tiangen). cDNA samples were used as templates to perform real‐time quantitative PCR with the power SYBR green mix (Abm, Cat.no. MasterMix‐LR) on an Applied Biosystems QuantStudio (TM) 7 Flex System (Applied Biosystems). Duplicate runs of each sample were normalized to 18S to determine the relative transcript abundance of target genes.

RT‐qPCR primer sequences as follows (F, forward; R: reverse): *18S*‐F: 5′‐AGTCCCTGCCCTTTGTACACA‐3′, *18S*‐R: 5′‐CGATCCGAGGGCCTCACTA‐3′; *Eif2α‐*F: 5′‐GATTTGTCAAAAAGAAGAGTTTC‐3′, *Eif2α‐*R: 5′‐TTGGATTTTGTGAATTTGTCCTC‐3′; *HSPA5‐*F: 5′‐ACTTGGGGACCACCTATTCCT‐3′, *HSPA5‐*R: 5′‐ATCGCCAATCAGACGCTCC‐3′; *Ddit3*‐F: 5′‐CTGGAAGCCTGGTATGAGGAT‐3′, *Ddit3‐*R: 5′‐CAGGGTCAAGAGTAGTGAAGGT‐3′, *Pink1‐*F: 5′‐TTCTTCCGCCAGTCGGTAG‐3′, *Pink1‐*R: 5′‐CTGCTTCTCCTCGATCAGCC‐3′. *Atf4‐*F: 5′‐CTCTTGACCACGTTGGATGAC‐3′, *Atf4‐*R: 5′‐CAACTTCACTGCCTAGCTCTAAA‐3′. *Erdj4‐*F: 5′‐AGGGAAGGATGAGGAAATCG‐3′, *Erdj4‐*R: 5′‐ACTGTTGTTGCCGTTTGG‐3′, *Gadd45a‐*F: 5′‐CCGAAAGGATGGACACGGTG‐3′, *Gadd45a‐*R: 5′‐TTATCGGGGTCTACGTTGAGC‐3′. *Nrf2‐*F: 5′‐CAACTCGGCGAAGAAAGAAACA‐3′, *Nrf2‐*R: 5′‐CAACTCGGCGAAGAAAGAAACA‐3′.

### 4.4 Protein extraction and western blot analysis

Operation of tissues: Tissues were placed in liquid nitrogen when mice were sacrificed and then would be stored in a −80°C refrigerator. A small piece of liver would be cut off and used for protein lysate extraction. A whole muscle from one leg of mice would be used for protein lysate extraction. Just half of the heart and brain would be used for protein lysate extraction. All tissue was extracted in 1 mL RIPA‐lysis buffer.

Protein was quantified by the method of BCA reagent‐chromogenic quantification. Tissue samples in RIPA buffer were homogenized by a homogenizer. Using the 1 mg/mL BSA protein standard, eight protein concentration gradients were set to plot the protein concentration curve. Dilute the sample to the appropriate dilution. The copper ion solution was mixed with the BCA solution at a volume ratio of 1:50 to prepare the BCA reaction working fluid. Add 200 μL reaction working fluid to the configured standard and diluted samples for testing. After incubation at 37°C for 15 min, the light absorption value at 562 nm was collected. The protein concentration of the target sample was calculated by using the concentration calculation equation generated by the standard product.

Total tissue lysates were extracted from tissues with ice‐cold radio‐immunoprecipitation assay (RIPA) lysis buffer (50 mM Tris pH 7.5, 150 mM NaCl, 0.1% SDS, 1% NP‐40, 0.5% sodium deoxycholate) supplemented with protease and phosphatase inhibitor cocktails (Pierce, Cat.no. 78441). The lysates were clarified by centrifugation at 13,000 rpm for 15 min, and protein concentrations were quantified by BCA (Solarbio, Cat.no. PC0021). Equal amounts (50 μg) of tissue lysates were resolved by SDS‐PAGE, blotted onto a nitrocellulose membrane (Pall, Cat.no. 66485), blocked with 5% milk in TBST for 1 h at room temperature, and incubated overnight with primary antibodies at 4°C. The blots were then washed and incubated with HRP‐conjugated secondary antibody for 1 h at room temperature and visualized by ECL (Merck Millipore, Cat.no. WBKL50500) using a ChemiDoc MP imaging system (BioRad). For the detection of phosphoepitopes, BSA instead of milk was used for blocking and antibody preparation.

### 4.5 Polysome profiling of liver

A linear sucrose gradient of 15%–45% (10 mM Hepes, pH 7.4, 75 mM KCI, 5 mM MgC1_2_, 100 μg/mL CHX, Sucrose) was prepared by the Gradient Master and then keep the gradient at 4°C for at least 30 min on the day before use. Cut 0.5 g fresh liver, put into cold wash buffer (250 mM sucrose, 1 mM MgCl2, 100 μg/mL CHX), and cut the tissue into small pellets to wash fully, then transfer the pellets to 1.5 mL Lysis (50 mM Hepes, pH 7.4, 75 mM KCI, 5 mM MgC1_2_, 250 mM sucrose,100 μg/mL CHX) buffer and homogenize the tissue using 2.8 × 10 (Steffen & Dillin, 2016) rpm, 0.3 min (XHF‐DY#, Ningbo SCIENTA). Centrifuge the homogenate at 3000 rpm for 5 min to discard the unbroken tissue. Transfer 1 mL supernatant to 1.5 mL RNase‐free EP tube, add 125 μL 10% Triton X‐100 and 125 μL 10% sodium deoxycholate, and leave on ice for 10 min. Centrifuge the homogenate at 13000 rpm for 10 min. Transfer 1 mL supernatant to the new 1.5 mL EP tube, mix well, and carefully layer 300 μL lysates on the top of the sucrose gradient‐centrifuge at 40,000 rpm for 2.5 h, then fraction and analysis.

### 4.6 Cell culture, transfection

HEK293A, HeLa, and C2C12 cells were obtained from ATCC. Cells were cultured in DMEM containing 10% fetal bovine serum (FBS). Small interference RNA was transfected with RNAiMAX (Thermo Scientific) according to manufacturer recommendations. Without specification, all siRNAs were transfected for 24 h before analysis or further treatment. Refer to Section 4 for reagents and kits used in this study.

### 4.7 Translation rate measurement in vivo

To minimize variation, we subjected the mice to a 12‐h food withdrawal and administered a puromycin injection after a 2‐h refeeding period. For all in vivo protein synthesis measurements, mice were anesthetized with 0.4% pentobarbital sodium and given an intravenous injection of puromycin (0.04 mol g^−1^ body weight) dissolved in 100 μL PBS. Exactly 30 min post‐injection, tissues were extracted and frozen in liquid nitrogen for western blot analysis.

### 4.8 Mice body composition measurement in vivo

The body composition of the mice was assessed under full wakefulness using Echo MIR‐100 and A10 (United Well Biotechnology). The mice were placed in a testing chamber and subjected to measurement. To prevent errors, the test chamber was washed with ddH_2_O after each measurement and dried.

### 4.9 Mice blood glucose test and ITT/GTT test

Glucose tolerance tests (GTT) were performed in overnight‐fasted mice by intraperitoneal glucose injection (1.5 g kg^−1^ body weight), and insulin tolerance tests (ITT) were performed by intraperitoneal insulin injection (1.2 IU kg^−1^ for young mice/ 1 IU kg^−1^ for aging mice) after a 6 h food withdrawal. Tail plasma glucose concentrations were measured before and after the injection at the indicated time points.

### 4.10 Triglyceride and cholesterol quantification

Whole blood samples were collected and centrifuged at 13,000 rpm for 30 min at 4°C. Triglyceride (STA‐396, Cell Biolabs) and cholesterol (STA‐384, Cell Biolabs) levels in serum or liver tissue were measured using a colorimetric method following the manufacturer's instructions.

### 4.11 Serum cytokine quantification

For tumor necrosis factor‐α (TNF‐α) and Interleukin 6 (IL‐6) measurements, whole blood samples were centrifuged for 30 min at 13,000 × *g*, 4°C to remove blood cells and debris, and measured by mouse TNF ELISA Set (BD Biosciences, Cat.no. 555268) and mouse IL‐6 ELISA Set (BD Biosciences, Cat.no. 555240), according to the manufacturer's instructions.

### 4.12 Metabolic cage studies

Continuous monitoring of whole‐body metabolism in mice was performed using the LabMaster Calorimetry System (TSE Systems, Germany), which measured parameters such as O_2_ and CO_2_ levels, temperature, food, and water intake, as well as physical activity in *x*/*y*/*z* planes. Mice were provided ad libitum access to a standard chow diet with sterile drinking water. Data were collected at 27‐min intervals over 3 days.

### 4.13 Seahorse XF96e analyzer measurement of mitochondria respiration

Primary hepatocytes were seeded at a density of 40,000 cells/well into 0.1% gelatin (Ameresco, Cat.no. 9704) coated XF 96‐well microplates. After a 4 h incubation at 37°C with 5% CO_2_, cells were washed and used for the following Seahorse measurement. For standard mitochondria function assessment, MitoStress analyses were carried out with a sequential injection of 4 mM oligomycin (OM), 0.5 mM Carbonyl cyanide‐4‐(trifluoromethoxy) phenylhydrazone (FCCP), and 2 mM Antimycin A/2 mM Rotenone (A/R) at final concentrations. Respiration and acidification rates were normalized to cell number.

### 4.14 Statistical analysis

Data are presented as mean ± SEM. Student's unpaired *t*‐test was performed to determine statistical differences between the two groups. *p*‐values of less than 0.05 were considered significant, and *p*‐values ranging from 0.05 to 0.2 were labeled on the graphs. N denotes the number of biological replicates in each experiment, and it is provided in the corresponding figure legends. Immunoblot quantification was completed by AlphaView (FluroChem FC3). All graphs were drawn in Graphpad Prism 8.0.2 software.

## AUTHOR CONTRIBUTIONS

H.H. conceived and designed this project. H.H. performed most experiments from polyribosome profiling, translation flux analysis, biochemical analysis, and animal studies; L.N. assisted in polyribosome profiling and animal studies. L.Y. assisted in seahorse analyses; Q.X. assisted in western blot and cell line experiments; Y.L., H.J. supervised the work. H.H. wrote the manuscript. L.C., H.J. reviewed/edited the manuscript.

## FUNDING INFORMATION

The work is supported by National Key Research and Development Program of China (2016YFA0502002 and 2017YFA0504603), National Natural Science Foundation of China (NSFC 31671229 and 81471072).

## CONFLICT OF INTEREST STATEMENT

The authors declare no conflicts of interests.

## Supporting information


Appendix S1.

## Data Availability

The data that support the findings of this study are available from the corresponding author upon reasonable request.

## References

[acel14348-bib-0001] Adomavicius, T. , Guaita, M. , Zhou, Y. , Jennings, M. D. , Latif, Z. , Roseman, A. M. , & Pavitt, G. D. (2019). The structural basis of translational control by eIF2 phosphorylation. Nature Communications, 10(1), 2136. 10.1038/s41467-019-10167-3 PMC6513899 31086188

[acel14348-bib-0002] Amorim, J. A. , Coppotelli, G. , Rolo, A. P. , Palmeira, C. M. , Ross, J. M. , & Sinclair, D. A. (2022). Mitochondrial and metabolic dysfunction in ageing and age‐related diseases. Nature Reviews. Endocrinology, 18(4), 243–258. 10.1038/s41574-021-00626-7 PMC9059418 35145250

[acel14348-bib-0003] Anisimova, A. S. , Alexandrov, A. I. , Makarova, N. E. , Gladyshev, V. N. , & Dmitriev, S. E. (2018). Protein synthesis and quality control in aging. Aging, 10(12), 4269–4288. 10.18632/aging.101721 30562164 PMC6326689

[acel14348-bib-0004] Baker, B. M. , Nargund, A. M. , Sun, T. , & Haynes, C. M. (2012). Protective coupling of mitochondrial function and protein synthesis via the eIF2α kinase GCN‐2. PLoS Genetics, 8(6), e1002760. 10.1371/journal.pgen.1002760 22719267 PMC3375257

[acel14348-bib-0005] Birkenfeld, A. L. , Lee, H. Y. , Majumdar, S. , Jurczak, M. J. , Camporez, J. P. , Jornayvaz, F. R. , Frederick, D. W. , Guigni, B. , Kahn, M. , Zhang, D. , Weismann, D. , Arafat, A. M. , Pfeiffer, A. F. , Lieske, S. , Oyadomari, S. , Ron, D. , Samuel, V. T. , & Shulman, G. I. (2011). Influence of the hepatic eukaryotic initiation factor 2alpha (eIF2alpha) endoplasmic reticulum (ER) stress response pathway on insulin‐mediated ER stress and hepatic and peripheral glucose metabolism. The Journal of Biological Chemistry, 286(42), 36163–36170. 10.1074/jbc.M111.228817 21832042 PMC3196114

[acel14348-bib-0006] Brown, M. K. , Chan, M. T. , Zimmerman, J. E. , Pack, A. I. , Jackson, N. E. , & Naidoo, N. (2014). Aging‐induced endoplasmic reticulum stress alters sleep and sleep homeostasis. Neurobiology of Aging, 35(6), 1431–1441. 10.1016/j.neurobiolaging.2013.12.005 24444805 PMC4019391

[acel14348-bib-0007] Chalil, S. , Pierre, N. , Bakker, A. D. , Manders, R. J. , Pletsers, A. , Francaux, M. , Klein‐Nulend, J. , Jaspers, R. T. , & Deldicque, L. (2015). Aging related ER stress is not responsible for anabolic resistance in mouse skeletal muscle. Biochemical and Biophysical Research Communications, 468(4), 702–707. 10.1016/j.bbrc.2015.11.019 26551463

[acel14348-bib-0008] Chen, T. , Takrouri, K. , Hee‐Hwang, S. , Rana, S. , Yefidoff‐Freedman, R. , Halperin, J. , Natarajan, A. , Morisseau, C. , Hammock, B. , Chorev, M. , & Aktas, B. H. (2013). Explorations of substituted urea functionality for the discovery of new activators of the heme‐regulated inhibitor kinase. Journal of Medicinal Chemistry, 56(23), 9457–9470. 10.1021/jm400793v 24261904 PMC3938169

[acel14348-bib-0009] Chen, Z. , Chen, Z. , & Jin, X. (2023). Mendelian randomization supports causality between overweight status and accelerated aging. Aging Cell, 22(8), e13899. 10.1111/acel.13899 37277933 PMC10410004

[acel14348-bib-0010] Chiocchetti, A. , Zhou, J. , Zhu, H. , Karl, T. , Haubenreisser, O. , Rinnerthaler, M. , Heeren, G. , Oender, K. , Bauer, J. , Hintner, H. , Breitenbach, M. , & Breitenbach‐Koller, L. (2007). Ribosomal proteins Rpl10 and Rps6 are potent regulators of yeast replicative life span. Experimental Gerontology, 42(4), 275–286. 10.1016/j.exger.2006.11.002 17174052

[acel14348-bib-0011] Conn, C. S. , Yang, H. , Tom, H. J. , Ikeda, K. , Oses‐Prieto, J. A. , Vu, H. , Oguri, Y. , Nair, S. , Gill, R. M. , Kajimura, S. , DeBerardinis, R. J. , Burlingame, A. L. , & Ruggero, D. (2021). The major cap‐binding protein eIF4E regulates lipid homeostasis and diet‐induced obesity. Nature Metabolism, 3(2), 244–257. 10.1038/s42255-021-00349-z PMC10350339 33619378

[acel14348-bib-0012] Das, I. , Krzyzosiak, A. , Schneider, K. , Wrabetz, L. , D'Antonio, M. , Barry, N. , Sigurdardottir, A. , & Bertolotti, A. (2015). Preventing proteostasis diseases by selective inhibition of a phosphatase regulatory subunit. Science (New York, N.Y.), 348(6231), 239–242. 10.1126/science.aaa4484 25859045 PMC4490275

[acel14348-bib-0013] Derisbourg, M. J. , Hartman, M. D. , & Denzel, M. S. (2021). Perspective: Modulating the integrated stress response to slow aging and ameliorate age‐related pathology. Nature Aging, 1(9), 760–768. 10.1038/s43587-021-00112-9 35146440 PMC7612338

[acel14348-bib-0014] Derisbourg, M. J. , Wester, L. E. , Baddi, R. , & Denzel, M. S. (2021). Mutagenesis screen uncovers lifespan extension through integrated stress response inhibition without reduced mRNA translation. Nature Communications, 12(1), 1678. 10.1038/s41467-021-21743-x PMC7960713 33723245

[acel14348-bib-0015] Drummond, D. A. , & Wilke, C. O. (2009). The evolutionary consequences of erroneous protein synthesis. Nature Reviews. Genetics, 10(10), 715–724. 10.1038/nrg2662 PMC2764353 19763154

[acel14348-bib-0016] Ellulu, M. S. , Patimah, I. , Khaza'ai, H. , Rahmat, A. , & Abed, Y. (2017). Obesity and inflammation: The linking mechanism and the complications. Archives of Medical Science, 13(4), 851–863. 10.5114/aoms.2016.58928 28721154 PMC5507106

[acel14348-bib-0017] Firpi, R. J. , Tran, T. T. , Flores, P. , Nissen, N. , Colquhoun, S. , Shackleton, C. , Martin, P. , Vierling, J. M. , & Poordad, F. F. (2004). Sirolimus‐induced hyperlipidaemia in liver transplant recipients is not dose‐dependent. Alimentary Pharmacology & Therapeutics, 19, 1033–1039. 15113371 10.1111/j.1365-2036.2004.01923.x

[acel14348-bib-0018] Gold, P. E. (2008). Protein synthesis inhibition and memory: Formation vs amnesia. Neurobiology of Learning and Memory, 89(3), 201–211. 10.1016/j.nlm.2007.10.006 18054504 PMC2346577

[acel14348-bib-0019] Hansen, M. , Taubert, S. , Crawford, D. , Libina, N. , Lee, S. J. , & Kenyon, C. (2007). Lifespan extension by conditions that inhibit translation in *Caenorhabditis elegans* . Aging Cell, 6, 95–110. 10.1111/j.1474-9726.2006.00267.x 17266679

[acel14348-bib-0020] Harding, H. P. , Zhang, Y. , Scheuner, D. , Chen, J. J. , Kaufman, R. J. , & Ron, D. (2009). Ppp1r15 gene knockout reveals an essential role for translation initiation factor 2 alpha (eIF2alpha) dephosphorylation in mammalian development. Proceedings of the National Academy of Sciences of the United States of America, 106(6), 1832–1837. 10.1073/pnas.0809632106 19181853 PMC2644123

[acel14348-bib-0021] Hetz, C. , & Saxena, S. (2017). ER, stress and the unfolded protein response in neurodegeneration. Nature Reviews. Neurology, 13(8), 477–491. 10.1038/nrneurol.2017.99 28731040

[acel14348-bib-0022] Hotamisligil, G. S. (2006). Inflammation and metabolic disorders. Nature, 444(7121), 860–867. 10.1038/nature05485 17167474

[acel14348-bib-0023] Jackson, R. J. , Hellen, C. U. , & Pestova, T. V. (2010). The mechanism of eukaryotic translation initiation and principles of its regulation. Nature Reviews. Molecular Cell Biology, 11(2), 113–127. 10.1038/nrm2838 20094052 PMC4461372

[acel14348-bib-0024] Janssens, G. E. , Molenaars, M. , Herzog, K. , Grevendonk, L. , Remie, C. M. E. , Vervaart, M. A. T. , Elfrink, H. L. , Wever, E. J. M. , Schomakers, B. V. , Denis, S. W. , Waterham, H. R. , Pras‐Raves, M. L. , van Weeghel, M. , van Kampen, A. H. C. , Tammaro, A. , Butter, L. M. , van der Rijt, S. , Florquin, S. , Jongejan, A. , … Houtkooper, R. H. (2024). A conserved complex lipid signature marks human muscle aging and responds to short‐term exercise. Nature Aging, 4, 681–693. 10.1038/s43587-024-00595-2 38609524

[acel14348-bib-0025] Jiang, H. Y. , Wek, S. A. , McGrath, B. C. , Scheuner, D. , Kaufman, R. J. , Cavener, D. R. , & Wek, R. C. (2003). Phosphorylation of the alpha subunit of eukaryotic initiation factor 2 is required for activation of NF‐kappaB in response to diverse cellular stresses. Molecular and Cellular Biology, 23(16), 5651–5663. 10.1128/MCB.23.16.5651-5663.2003 12897138 PMC166326

[acel14348-bib-0026] Johnston, O. , Rose, C. L. , Webster, A. C. , & Gill, J. S. (2008). Sirolimus is associated with new‐onset diabetes in kidney transplant recipients. Journal of the American Society of Nephrology, 19, 1411–1418. 18385422 10.1681/ASN.2007111202 PMC2440303

[acel14348-bib-0027] Kennedy, B. K. , Berger, S. L. , Brunet, A. , Campisi, J. , Cuervo, A. M. , Epel, E. S. , Franceschi, C. , Lithgow, G. J. , Morimoto, R. I. , Pessin, J. E. , Rando, T. A. , Richardson, A. , Schadt, E. E. , Wyss‐Coray, T. , & Sierra, F. (2014). Geroscience: Linking aging to chronic disease. Cell, 159(4), 709–713. 10.1016/j.cell.2014.10.039 25417146 PMC4852871

[acel14348-bib-0028] Kimball, S. R. (1999). Eukaryotic initiation factor eIF2. The International Journal of Biochemistry & Cell Biology, 31(1), 25–29. 10.1016/s1357-2725(98)00128-9 10216940

[acel14348-bib-0029] Kimball, S. R. , Fabian, J. R. , Pavitt, G. D. , Hinnebusch, A. G. , & Jefferson, L. S. (1998). Regulation of guanine nucleotide exchange through phosphorylation of eukaryotic initiation factor eIF2alpha. Role of the alpha‐ and delta‐subunits of eiF2b. The Journal of Biological Chemistry, 273(21), 12841–12845. 10.1074/jbc.273.21.12841 9582312

[acel14348-bib-0030] Klaips, C. L. , Jayaraj, G. G. , & Hartl, F. U. (2018). Pathways of cellular proteostasis in aging and disease. The Journal of Cell Biology, 217(1), 51–63. 10.1083/jcb.201709072 29127110 PMC5748993

[acel14348-bib-0031] Kourtis, N. , & Tavernarakis, N. (2011). Cellular stress response pathways and ageing: Intricate molecular relationships. The EMBO Journal, 30(13), 2520–2531. 10.1038/emboj.2011.162 21587205 PMC3155297

[acel14348-bib-0032] Kraig, E. , Linehan, L. A. , Liang, H. , Romo, T. Q. , Liu, Q. , Wu, Y. , Benavides, A. D. , Curiel, T. J. , Javors, M. A. , Musi, N. , Chiodo, L. , Koek, W. , Gelfond, J. A. L. , & Kellogg, D. L., Jr. (2018). A randomized control trial to establish the feasibility and safety of rapamycin treatment in an older human cohort: Immunological, physical performance, and cognitive effects. Experimental Gerontology, 105, 53–69. 10.1016/j.exger.2017.12.026 29408453 PMC5869166

[acel14348-bib-0033] Krueger, D. A. , Wilfong, A. A. , Mays, M. , Talley, C. M. , Agricola, K. , Tudor, C. , Capal, J. , Holland‐Bouley, K. , & Franz, D. N. (2016). Long‐term treatment of epilepsy with everolimus in tuberous sclerosis. Neurology, 87, 2408–2415. 27815402 10.1212/WNL.0000000000003400 PMC5177677

[acel14348-bib-0034] Krzyzosiak, A. , Sigurdardottir, A. , Luh, L. , Carrara, M. , Das, I. , Schneider, K. , & Bertolotti, A. (2018). Target‐based discovery of an inhibitor of the regulatory phosphatase PPP1R15B. Cell, 174(5), 1216–1228. 10.1016/j.cell.2018.06.030 30057111 PMC6108835

[acel14348-bib-0035] Kulalert, W. , & Kim, D. H. (2013). The unfolded protein response in a pair of sensory neurons promotes entry of *C*. *Elegans* into dauer diapause. Current Biology: CB, 23(24), 2540–2545. 10.1016/j.cub.2013.10.058 24316205 PMC3870035

[acel14348-bib-0036] Kulalert, W. , Sadeeshkumar, H. , Zhang, Y. K. , Schroeder, F. C. , & Kim, D. H. (2017). Molecular determinants of the regulation of development and metabolism by neuronal eIF2α phosphorylation in *Caenorhabditis elegans* . Genetics, 206(1), 251–263. 10.1534/genetics.117.200568 28292919 PMC5419473

[acel14348-bib-0037] Kyriakakis, E. , Princz, A. , & Tavernarakis, N. (2015). Stress responses during ageing: Molecular pathways regulating protein homeostasis. Methods in Molecular Biology (Clifton, N.J.), 1292, 215–234. 10.1007/978-1-4939-2522-3_16 25804759

[acel14348-bib-0038] Ladiges, W. , Morton, J. , Blakely, C. , & Gale, M. (2000). Tissue specific expression of PKR protein kinase in aging B6D2F1 mice. Mechanisms of Ageing and Development, 114(2), 123–132. 10.1016/s0047-6374(00)00097-x 10799709

[acel14348-bib-0039] Li, Q. , & Hoppe, T. (2023). Role of amino acid metabolism in mitochondrial homeostasis. Frontiers in Cell and Developmental Biology, 11, 1127618. 10.3389/fcell.2023.1127618 36923249 PMC10008872

[acel14348-bib-0040] Mac Callum, P. E. , Hebert, M. , Adamec, R. E. , & Blundell, J. (2014). Systemic inhibition of mTOR kinase via rapamycin disrupts consolidation and reconsolidation of auditory fear memory. Neurobiology of Learning and Memory, 112, 176–185. 10.1016/j.nlm.2013.08.014 24012802

[acel14348-bib-0041] Mannick, J. B. , & Lamming, D. W. (2023). Targeting the biology of aging with mTOR inhibitors. Nature Aging, 3(6), 642–660. 10.1038/s43587-023-00416-y 37142830 PMC10330278

[acel14348-bib-0042] Merrick, W. C. , & Pavitt, G. D. (2018). Protein synthesis initiation in eukaryotic cells. Cold Spring Harbor Perspectives in Biology, 10(12), a033092. 10.1101/cshperspect.a033092 29735639 PMC6280705

[acel14348-bib-0043] Mina, T. , Yew, Y. W. , Ng, H. K. , Sadhu, N. , Wansaicheong, G. , Dalan, R. , Low, D. Y. W. , Lam, B. C. C. , Riboli, E. , Lee, E. S. , Ngeow, J. , Elliott, P. , Griva, K. , Loh, M. , Lee, J. , & Chambers, J. (2023). Adiposity impacts cognitive function in Asian populations: An epidemiological and Mendelian randomization study. The Lancet Regional Health. Western Pacific, 33, 100710. 10.1016/j.lanwpc.2023.100710 36851942 PMC9957736

[acel14348-bib-0044] Moncan, M. , Mnich, K. , Blomme, A. , Almanza, A. , Samali, A. , & Gorman, A. M. (2021). Regulation of lipid metabolism by the unfolded protein response. Journal of Cellular and Molecular Medicine, 25(3), 1359–1370. 10.1111/jcmm.16255 33398919 PMC7875919

[acel14348-bib-0045] Oliveira, M. M. , Lourenco, M. V. , Longo, F. , Kasica, N. P. , Yang, W. , Ureta, G. , Ferreira, D. D. P. , Mendonça, P. H. J. , Bernales, S. , Ma, T. , De Felice, F. G. , Klann, E. , & Ferreira, S. T. (2021). Correction of eIF2‐dependent defects in brain protein synthesis, synaptic plasticity, and memory in mouse models of Alzheimer's disease. Science Signaling, 14(668), eabc5429. 10.1126/scisignal.abc5429 33531382 PMC8317334

[acel14348-bib-0046] Saxton, R. A. , & Sabatini, D. M. (2017). mTOR signaling in growth, metabolism, and disease. Cell, 168(6), 960–976. 10.1016/j.cell.2017.02.004 28283069 PMC5394987

[acel14348-bib-0047] Scheuner, D. , Song, B. , McEwen, E. , Liu, C. , Laybutt, R. , Gillespie, P. , Saunders, T. , Bonner‐Weir, S. , & Kaufman, R. J. (2001). Translational control is required for the unfolded protein response and in vivo glucose homeostasis. Molecular Cell, 7(6), 1165–1176. 10.1016/s1097-2765(01)00265-9 11430820

[acel14348-bib-0048] Schumacher, B. , Pothof, J. , Vijg, J. , & Hoeijmakers, J. H. J. (2021). The central role of DNA damage in the ageing process. Nature, 592(7856), 695–703. 10.1038/s41586-021-03307-7 33911272 PMC9844150

[acel14348-bib-0049] Skariah, G. , & Todd, P. K. (2021). Translational control in aging and neurodegeneration. Wiley Interdisciplinary Reviews: RNA, 12(4), e1628. 32954679 10.1002/wrna.1628 PMC7979572

[acel14348-bib-0050] Snieckute, G. , Ryder, L. , Vind, A. C. , Wu, Z. , Arendrup, F. S. , Stoneley, M. , Chamois, S. , Martinez‐Val, A. , Leleu, M. , Dreos, R. , Russell, A. , Gay, D. M. , Genzor, A. V. , Choi, B. S. , Basse, A. L. , Sass, F. , Dall, M. , Dollet, L. C. M. , Blasius, M. , … Bekker‐Jensen, S. (2023). ROS‐induced ribosome impairment underlies ZAKα‐mediated metabolic decline in obesity and aging. Science (New York, N.Y.), 382(6675), eadf3208. 10.1126/science.adf3208 38060659

[acel14348-bib-0051] Song, P. , Yang, F. , Jin, H. , & Wang, X. (2021). The regulation of protein translation and its implications for cancer. Signal Transduction and Targeted Therapy, 6(1), 68. 10.1038/s41392-020-00444-9 33597534 PMC7889628

[acel14348-bib-0052] Steffen, K. K. , & Dillin, A. (2016). A ribosomal perspective on proteostasis and aging. Cell Metabolism, 23(6), 1004–1012. 10.1016/j.cmet.2016.05.013 27304502

[acel14348-bib-0053] Syntichaki, P. , Troulinaki, K. , & Tavernarakis, N. (2007). eIF4E function in somatic cells modulates ageing in *Caenorhabditis elegans* . Nature, 445, 922–926. 10.1038/nature05603 17277769

[acel14348-bib-0054] Trelinska, J. , Dachowska, I. , Kotulska, K. , Fendler, W. , Jozwiak, S. , & Mlynarski, W. (2015). Complications of mammalian target of rapamycin inhibitor anticancer treatment among patients with tuberous sclerosis complex are common and occasionally life‐threatening. Anti‐Cancer Drugs, 26, 437–442. 25719621 10.1097/CAD.0000000000000207

[acel14348-bib-0055] Tsugawa, H. , Ishihara, T. , Ogasa, K. , Iwanami, S. , Hori, A. , Takahashi, M. , Yamada, Y. , Satoh‐Takayama, N. , Ohno, H. , Minoda, A. , & Arita, M. (2024). A lipidome landscape of aging in mice. Nature Aging, 4, 709–726. 10.1038/s43587-024-00610-6 38609525

[acel14348-bib-0056] Vellai, T. , Takacs‐Vellai, K. , Zhang, Y. , Kovacs, A. L. , Orosz, L. , & Müller, F. (2003). Genetics: influence of TOR kinase on lifespan in C. elegans. Nature, 426, 620. 10.1038/426620a 14668850

[acel14348-bib-0057] Way, S. W. , Podojil, J. R. , Clayton, B. L. , Zaremba, A. , Collins, T. L. , Kunjamma, R. B. , Robinson, A. P. , Brugarolas, P. , Miller, R. H. , Miller, S. D. , & Popko, B. (2015). Pharmaceutical integrated stress response enhancement protects oligodendrocytes and provides a potential multiple sclerosis therapeutic. Nature Communications, 6, 6532. 10.1038/ncomms7532 PMC4360920 25766071

[acel14348-bib-0058] Zhu, P. J. , Khatiwada, S. , Cui, Y. , Reineke, L. C. , Dooling, S. W. , Kim, J. J. , Li, W. , Walter, P. , & Costa‐Mattioli, M. (2019). Activation of the ISR mediates the behavioral and neurophysiological abnormalities in down syndrome. Science (New York, N.Y.), 366(6467), 843–849. 10.1126/science.aaw5185 31727829 PMC7299149

