# Supplementary material for: The translation initiation factor eIF2α regulates lipid homeostasis and metabolic aging
Source: Aging Cell. 2024 Oct 15;24(1):e14348. doi: 10.1111/acel.14348 (PMC11709108; doi:10.1111/acel.14348)
Supplement: Supplementary file 1 — Appendix S1. [file ACEL-24-e14348-s001.pdf]

Supplementary File

Title

The translation initiation factor eIF2α regulates lipid homeostasis and metabolic aging

10 pages in total

Figure S1-S7

Extended Data Figs

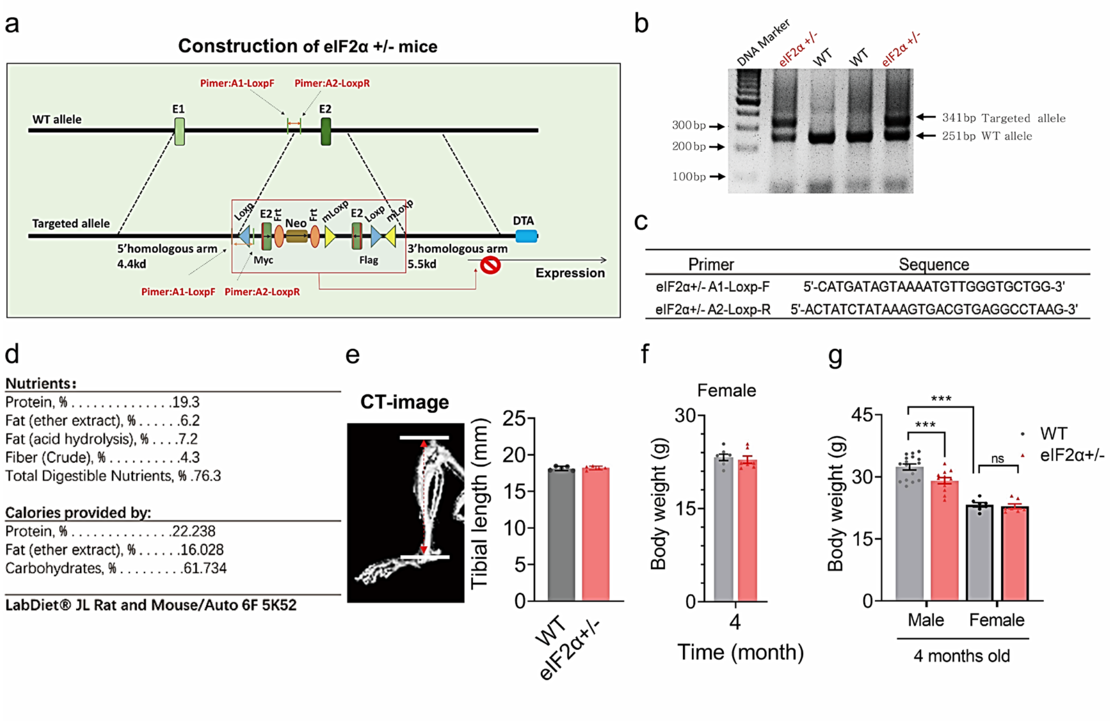

Fig S1. Construction of eIF2α+/- mice.

(a) Detailed schematic diagram at the position of gene *Eif2s1* in the genome of WT and eIF2α+/- mice. The insertion suppressed the expression of the targeted allele. **(b, c)** Genotyping of eIF2α+/- mice, PCR detection of targeted allele, and primers used. **(d)** Nutrient composition table of animal feed used in mouse feeding. **(e)** CT-imaging of mouse tibia and quantification of the length. **(f)** Body weight of WT (n=7) and eIF2α+/- (n=7) female mice at indicated age. **(g)** Body weight of male and female mice at the indicated age. Data are represented as mean ± SEM.

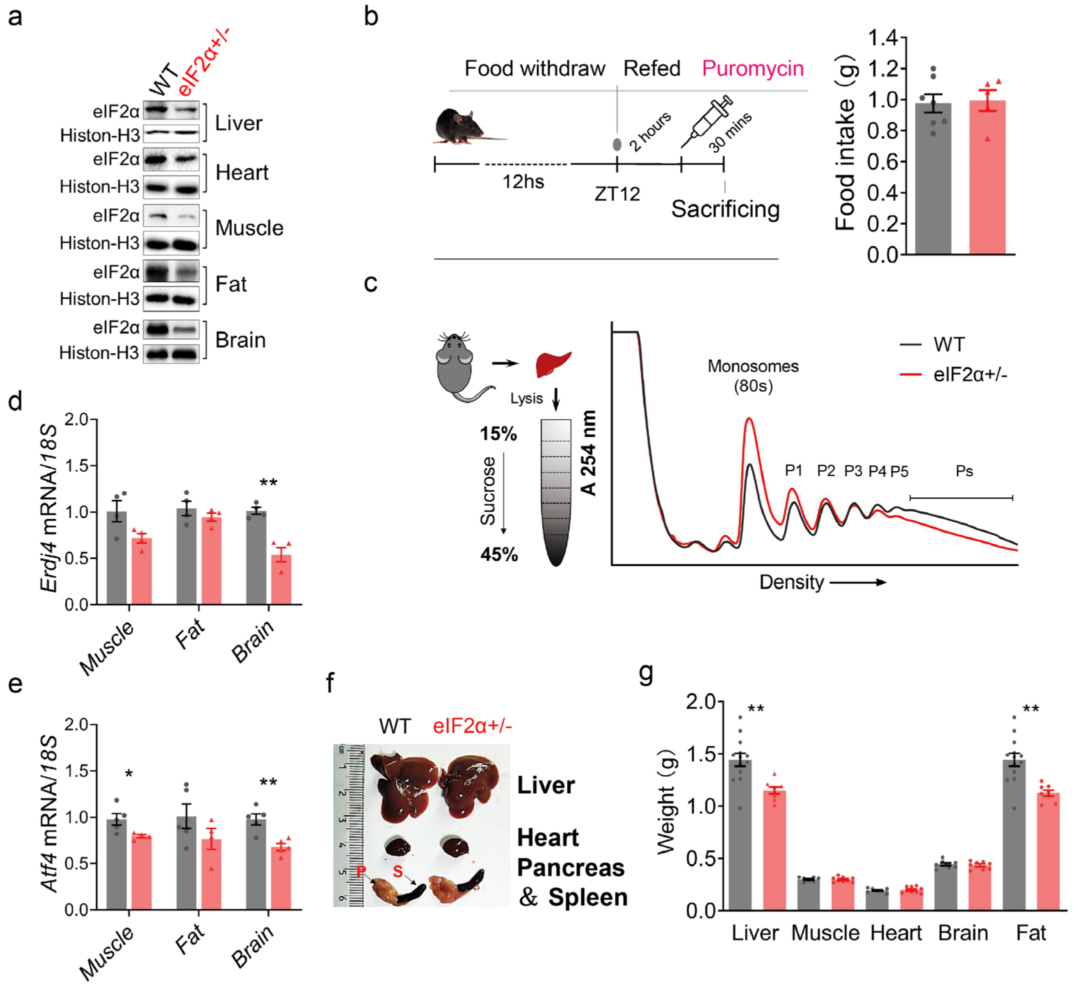

Fig S2. Downregulation of eIF2α in tissues inhibits protein synthesis.

(a) Representative western blot of eIF2α in multi-tissues. **(b)** Schematic flow of the translation labeling experiment, food intake of mice in the refed process. **(c)** Representative polysome profiles showing cytosolic polysome abundances in the liver of eIF2α+/- mice. Lysate is normalized to protein levels. The subunits (40S and 60S), monosomal peak (80S), and polysomal peak numbers are indicated (P1-P5 and Ps). **(d, e)** Measurement of the transcript levels of *Erdj4*, *Atf4* in multi-tissues. Normalized to *18S* ribosomal RNA (n = 4-5). **(f)** Tissue image of WT and eIF2α+/- mice, different tissues from the same mice in both groups. **(g)** Tissue weight measurement of mice at 4-month-old, WT (n=12~13) and eIF2α+/- (n=8~9). Data are represented as mean ± SEM. *p < 0.05, **p < 0.01.

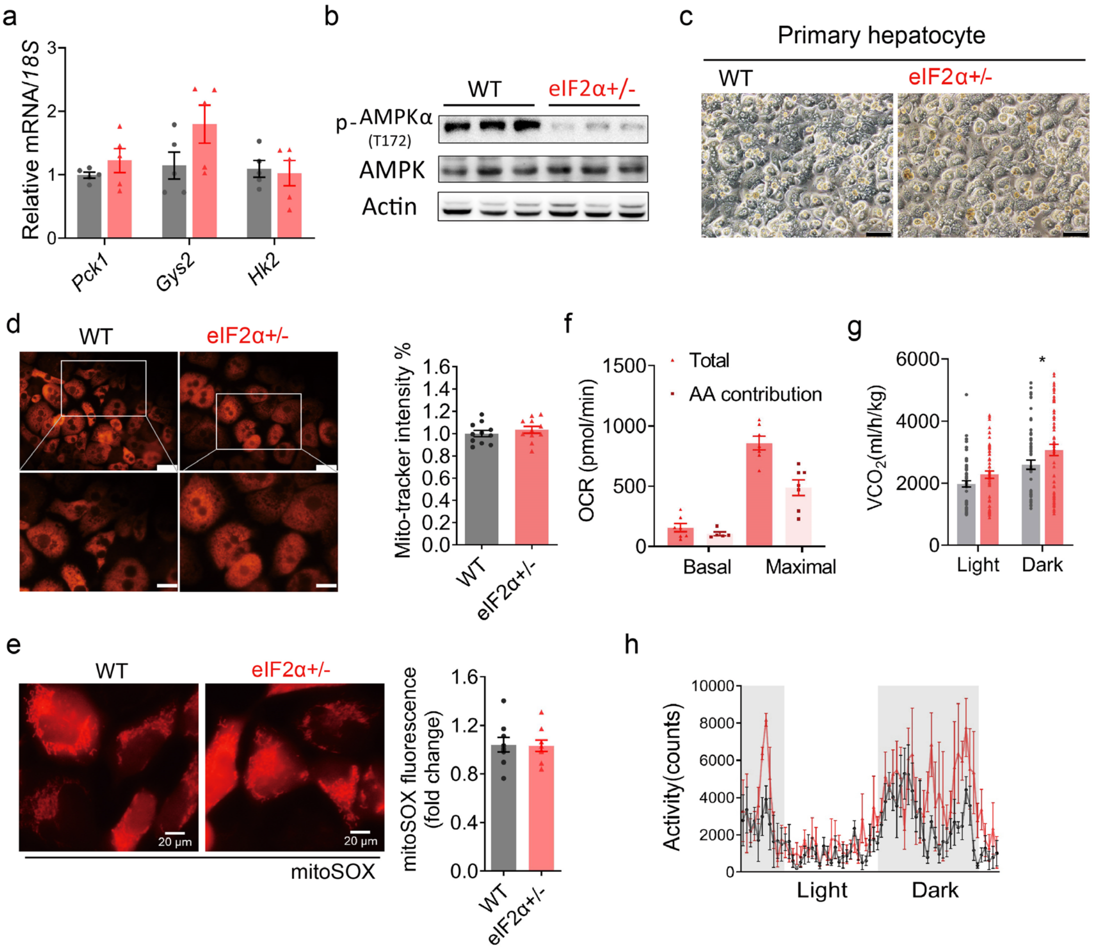

Fig S3. Detection of metabolic levels regulated by eIF2α.

(a) RT-qPCR examinations of glycolysis-related genes in the liver of WT (n=4~5) and eIF2α+/- (n=4~5) mice (4-month-old). **(b)** Western blot of AMPK pathway in the hepatocytes. **(c)** Representative images of cultured primary hepatocytes of WT and eIF2α+/- mice (Scale bar, 100 μm). **(d)** Mito-tracker marked mitochondrial in the primary hepatocyte, quantification of Mito-tracker intensity of per cell and normalized to the cell area, the relative intensity was normalized to the mean of WT group. (Scale bars, 50 μm of the top, 25 μm of the bottom). **(e)** ROS levels in primary hepatocytes of WT and eIF2α+/- mice were labeled using mitoSOX and fluorescence values per unit mitochondrial area (100 μm^2) (n=7) were quantified, and relative intensity was normalized to the mean of the WT group. **(f)** Mitochondrial respiration measurement of hepatocyte (4-month-old mice), OCR inhibited by AOA (inhibitor of the transaminase process) indicates amino acid (AA)-contributed respiration. **(g, h)** Metabolic cage

experiment, measurements of carbon dioxide production (g), motor activity (h), of WT and eIF2α+/- mice (4-month-old) (n=8 vs 8). Data are represented as mean ± SEM. *$p < 0.05$.

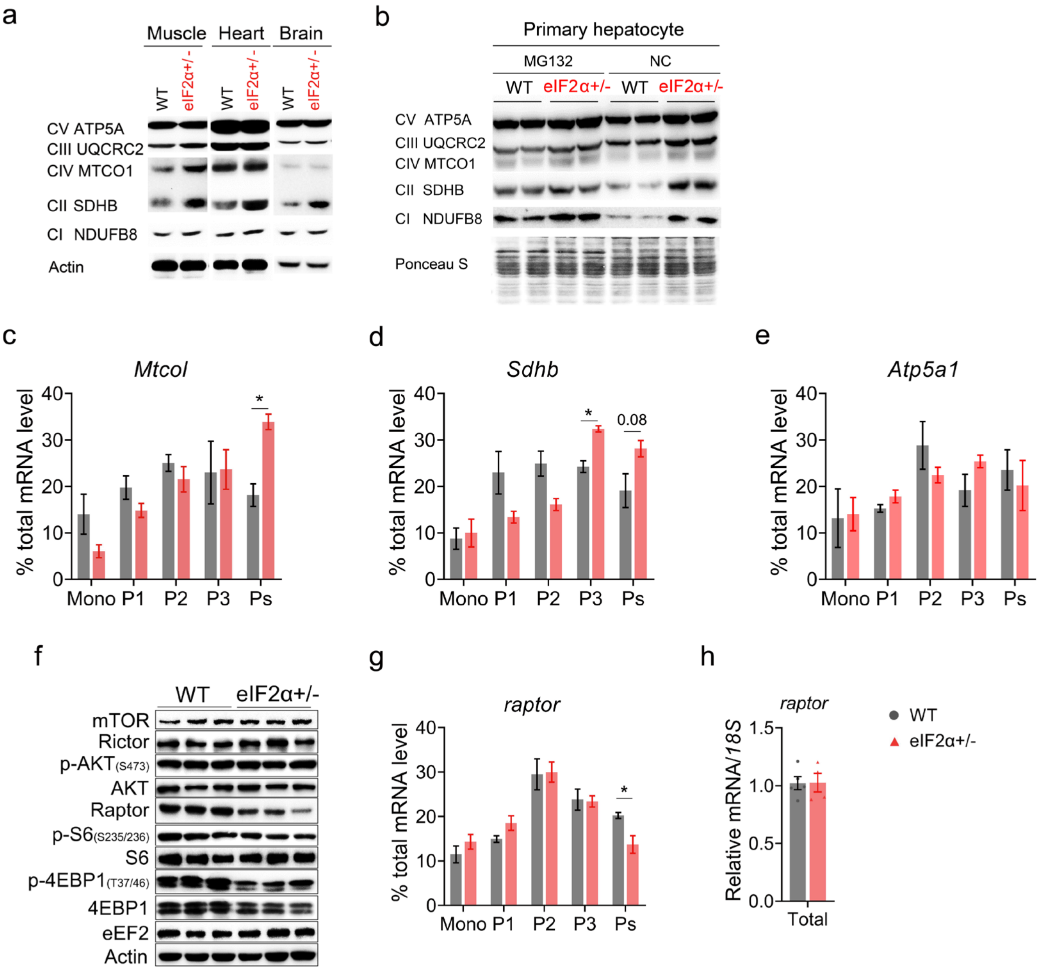

Fig S4. Translational regulation regulated by eIF2α.

(a) Representative western blot for ETC proteins in muscle, heart, and brain. **(b)** Western blot for ETC proteins in the primary hepatocytes of WT and eIF2α+/- mice. **(c-e)** The qPCR analysis of the *Mtcol, Sdhb, Atp5a1* in isolated sucrose gradient fractions of WT or eIF2α+/- livers. Normalized to *18S* ribosomal RNA (n = 3). **(f)** Western blot of mTOR pathways in the liver. **(g, h)** qPCR analysis of gradient fractions of *raptor* (g) and total mRNA analysis (h). Data are represented as mean ± SEM. *p < 0.05.

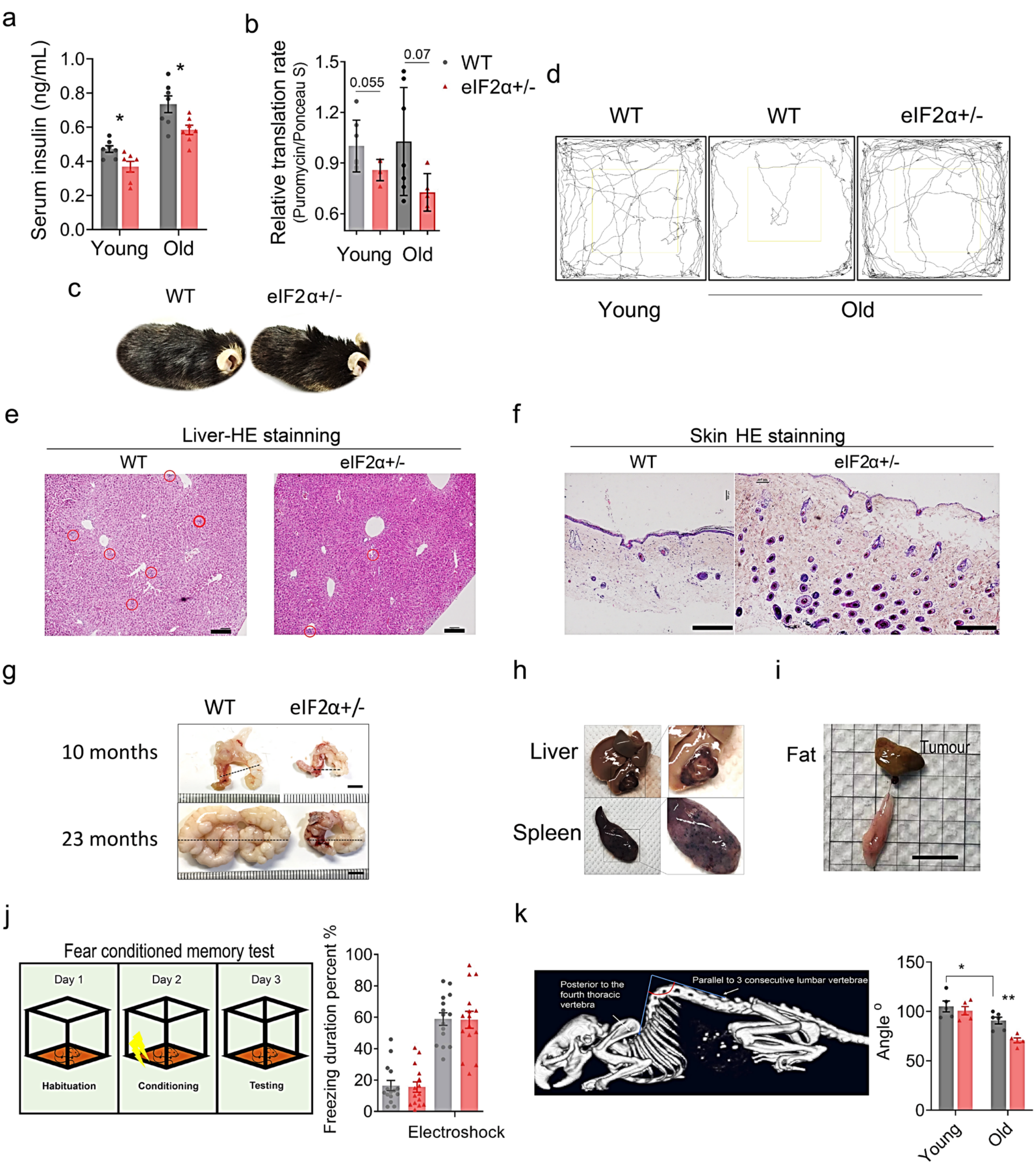

Fig S5. Lesions in multiple organs in aging.

(a) Measurement of serum insulin level (n=7 of each group). **(b)** Quantify translational flux in the liver of 24-month-old, WT (n=7) and eIF2α+/- (n=5) mice, the level of puromycin labeling was normalized to total protein. **(c)** Representative skin images of WT and eIF2α+/ mice (21-month-old). **(d)** Representative movement paths of mice in the Open field experiments. **(e)** Representative images of H&E staining liver sections (the cluster of immune cells were highlighted by the circle marks, Scale bar, 200 μm). **(f)** Skin and hair follicles, micrograph of a section through the skin, showing hair follicles (dark purple circular structures). Representative

images of H&E staining (Scale bar, 300 μm). **(g)** Seminal vesicle hypertrophy, schematic width measurement of WT and eIF2α+/- mice. **(h, i)** Tumor of in the liver, enlarged spleen (h), and lipoma (i) (Scale bar, 1 cm) in WT and eIF2α+/- in aging. **(j)** Schematic diagram of the fear-conditioned memory test, fear conditioned memory test of the WT (n=14) and eIF2α+/- (n=14) mice at the 22-month-old, fear memory remind test, freezing time (%) of mice during environmental acclimatization and electroshock experiments. **(k)** Schematic diagram of spinal curvature measurement in mice, spinal curvature was quantified respectively. Data are represented as mean ± SEM. $*p < 0.05$, $**p < 0.01$.

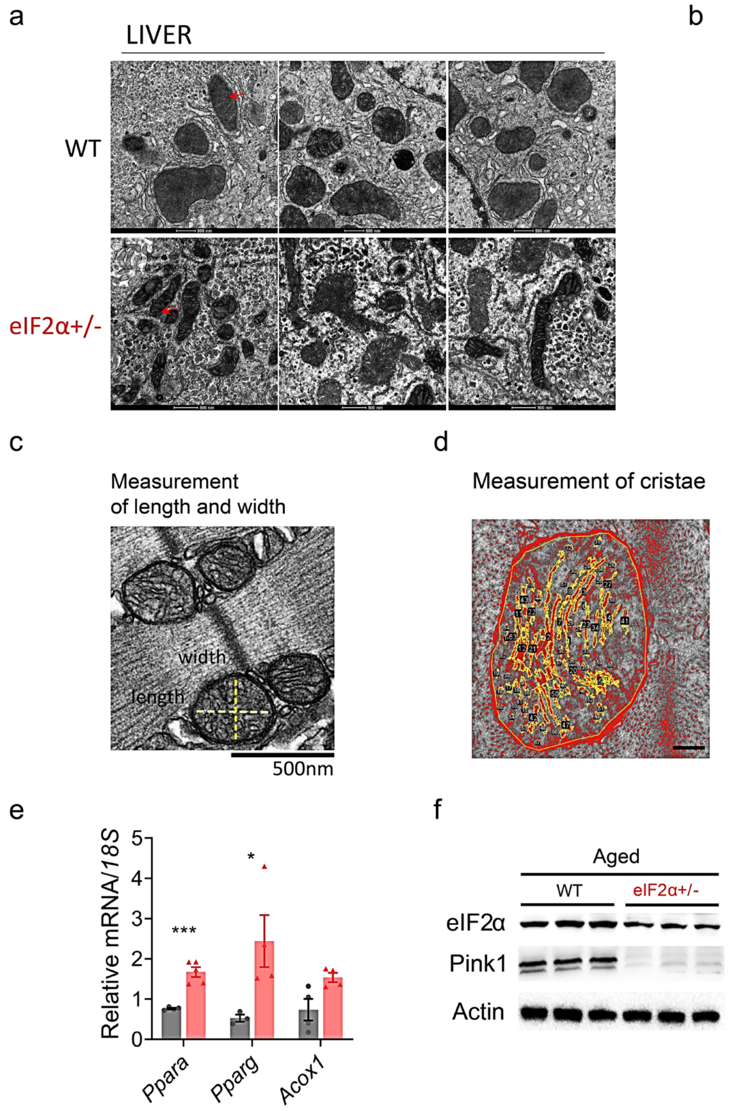

Fig S6. Pink1 level and graphical measurements on mitochondria.

(a-d) Ultrastructure of the liver (a), quantification of mitochondrial area (b), Scheme of measurements of mitochondrial length and width (Scale bar, 500 nm) (c), Quantification of mitochondrial cristae (Scale bar, 200 nm) (d), imaging data were processed and quantified with ImageJ (1.49V). **(e)** RT-qPCR examinations of gene expression, fatty acid oxidation (FAO) related genes of WT (n=4~5) and eIF2α+/- (n=4~5) mice (24-month-old). **(f)** Western blot of Pink1 in the liver of aging mice. Data are represented as mean ± SEM. *$p < 0.05$, ***$p < 0.001$.

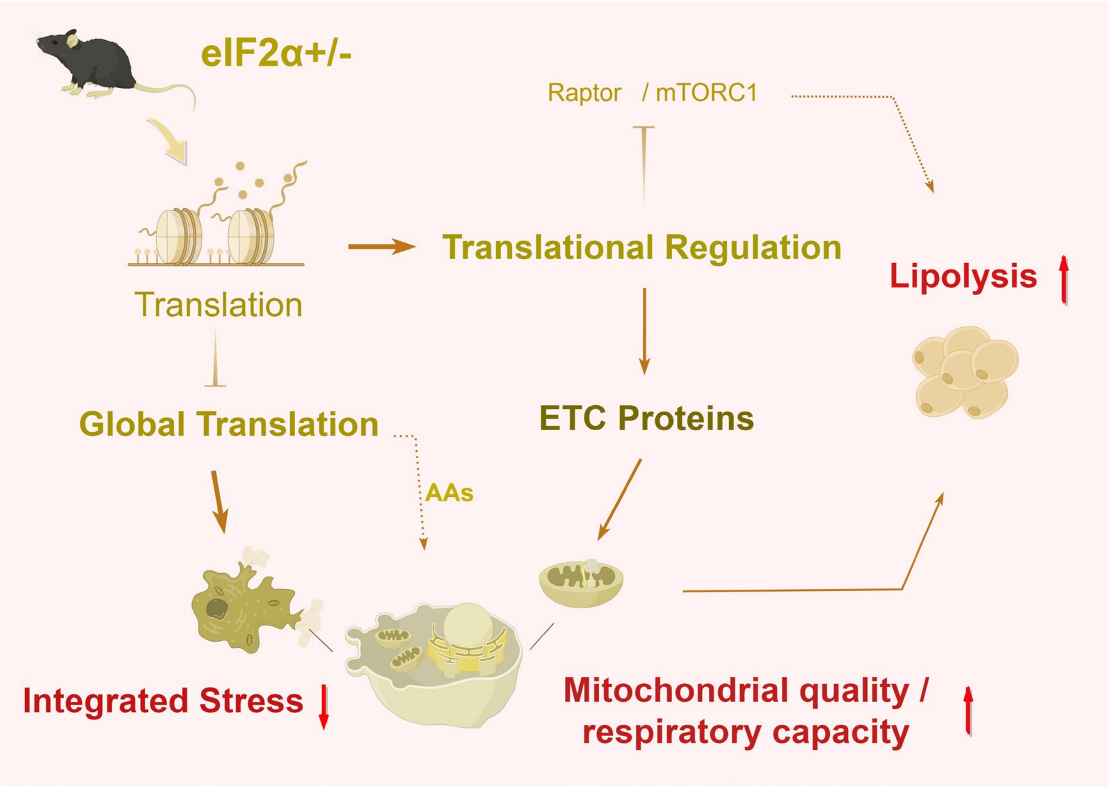

Fig S7. Schematic overview of the molecular regulatory mechanism in eIF2α+/- mice.

Suppression of global translation through reduced eIF2α induces the release of integrated stress, and translational regulation of ETC proteins and Raptor. Lower protein synthesis flux makes more AAs available to mitochondria, in conjunction with a more robust respiratory chain, contributes to an elevation in mitochondrial respiration, thereby promoting fat burning.
